# Can verbal suggestions strengthen the effects of a relaxation intervention?

**DOI:** 10.1371/journal.pone.0220112

**Published:** 2019-08-07

**Authors:** Lemmy Schakel, Dieuwke S. Veldhuijzen, Henriët van Middendorp, Meriem Manaï, Stefanie H. Meeuwis, Pieter Van Dessel, Andrea W. M. Evers

**Affiliations:** 1 Faculty of Social and Behavioural Sciences, Institute of Psychology, Health, Medical and Neuropsychology Unit, Leiden University, Leiden, The Netherlands; 2 Leiden Institute for Brain and Cognition, Leiden University, Leiden, the Netherlands; 3 Department of Experimental Clinical and Health Psychology, Ghent University, Ghent, Belgium; 4 Department of Psychiatry, Leiden University Medical Centre, Leiden, The Netherlands; Maastricht Universitair Medisch Centrum+, NETHERLANDS

## Abstract

Short stress management interventions such as relaxation therapy have demonstrated preliminary effectiveness in reducing stress-related problems. A promising tool to strengthen the effectiveness of relaxation-based interventions is the use of verbal suggestions, as previous research provided evidence that verbal suggestions can induce positive outcome expectancies, facilitate adaptive responses to stress and improve health outcomes. The present experimental proof-of-concept study aimed to investigate the effects of a brief relaxation intervention and specifically the role of verbal suggestions on stress-related outcomes assessed by self-report questionnaires and psychophysiological data. 120 participants (*mean* age = 22.1 years) were randomized to one of four intervention conditions: a brief relaxation intervention plus verbal suggestions condition, a brief relaxation intervention only condition, a verbal suggestions only condition, and a control condition. Afterwards, participants were subjected to a psychosocial stress challenge to assess reactivity to a stressful event. Immediately after both relaxation interventions (with and without verbal suggestions), lower self-reported state anxiety was found compared to the control condition, but no differences were observed in response to the stressor. The verbal suggestions only condition did not impact state anxiety. No significant effects were found for verbal suggestion interventions on cortisol, alpha amylase, heart rate and skin conductance. This is the first study investigating the role of verbal suggestions in the effectiveness of a brief relaxation intervention. Although this experimental proof-of-concept study provides support for the effectiveness of a brief relaxation intervention in lowering state anxiety directly after the intervention, the effects did not impact the response to a subsequent stressor and we did not observe any evidence for the add-on effectiveness of verbal suggestions. The effectiveness of brief relaxation interventions on stress responses should be investigated further in future research by incorporating interventions that are tailored to the specific stress challenge and various types of verbal suggestions.

## Introduction

Accumulating findings demonstrate that intense acute stress and prolonged experienced stress can have adverse effects on health [1, 2]. Stress management interventions have demonstrated to be effective in reducing stress and are considered beneficial for health [3, 4]. Relaxation therapy, i.e., learning to focus on arousal reduction by releasing muscle tension [5], is one of the most commonly used types of stress management interventions [4, 6]. Most relaxation interventions involve multiple sessions, but there is also some initial support for the effectiveness of brief (2 to 3 sessions) interventions [7, 8]. One study even found preliminary evidence for the effectiveness of a relaxation intervention during one single session of 15–20 minutes in reducing acute distress [9]. To improve the effectiveness of relaxation-based interventions, it might be useful to investigate potential factors that influence the effects of brief interventions for the treatment of stress consequences.

Verbal suggestions are a promising tool to strengthen the effectiveness of relaxation-based interventions, as it has been argued that expectancies towards the outcomes of interventions, e.g., expecting beforehand that an intervention will result in a reduction of stress, might strongly moderate the effects of (stress management) interventions. Because verbal suggestions are thought to provide a good way to steer outcome expectancies [10], they might improve outcomes of relaxation-based interventions. The effectiveness of verbal suggestions is not yet investigated in the context of stress management interventions. However, it has already been shown that verbal suggestions by themselves can alter stress responses. Two experimental studies showed that verbal suggestions regarding biofeedback responses (i.e., informing participants that they have a high heartbeat and are aroused, or that they have a calm heartbeat and are relaxed) altered their psychophysiological stress responses, in that participants became more aroused after being provided with verbal suggestions [11, 12]. In addition, studies in the placebo literature have provided strong evidence that verbal suggestions can result in symptom relief in several psychiatric and non-psychiatric conditions, as well as somatic conditions [13, 14]. For instance, a study of Skvortsova and colleagues (2018) found that verbal suggestions can reduce levels of self-reported pain [15]. Since enhanced outcome expectancies through verbal suggestions can influence psychobiological stress responses as well as various somatic symptoms, they may possibly also effectively optimize the effectiveness of a short stress management intervention. We therefore investigated whether verbal suggestions can strengthen the effects of a relaxation intervention by adding those verbal suggestions onto the relaxation intervention.

The present experimental proof-of-concept study investigates the effects of a brief relaxation intervention based on a single session on stress-related outcomes and more specifically assesses whether verbal suggestions can optimize these effects. Participants between 18 and 35 years of age were randomized to one of four conditions: a brief relaxation intervention plus verbal suggestions condition, a brief relaxation intervention only condition, a verbal suggestions only condition, and a control condition without any relaxation practice or verbal suggestions. As the aim of the brief relaxation intervention and the verbal suggestions is to optimize coping with everyday life stressors, it is interesting to investigate the effectiveness of these interventions on stress-related outcomes by exposing people to a well-validated real-life psychosocial stress challenge and subsequently evaluate the stress response. We therefore administered a validated stress challenge test to all participants. First of all, we examined whether participants in the brief relaxation intervention conditions (with or without verbal suggestions) would show less self-reported state anxiety immediately after the intervention, as well as directly and multiple time points after the stress challenge, compared to the control condition. In case of significant group differences, it was furthermore examined whether (1) the brief relaxation intervention plus verbal suggestions condition outperforms the brief relaxation intervention only condition, and (2) the verbal suggestions only condition outperforms the control condition. Subsequently, we studied effects for secondary self-reported and psychophysiological outcomes, including self-reported well-being, self-reported positive and negative affect, salivary cortisol and alpha-amylase, as well as heart rate and skin conductance.

## Methods

### Ethics statement

The protocol was approved by the local psychological ethics committee of Leiden University (registration code: CEP17-0102/1). Preregistration of the study was done at the Netherlands Trial Register (registration code: NTR6392) and the study was performed according to the Declaration of Helsinki (2013).

### Design

The present study used a randomized experimental study design. Based on a 1:1:1:1 allocation ratio, participants were randomized to one of the four conditions (see above), stratified for gender. The randomization procedure was based on block randomization (block size = 12), generated by an online random number generator (www.random.org). Since stratification of gender was determined over blocks and not specifically over conditions, this resulted in nonsignificant differences in number of females and males over groups. During the experiment, participants were blinded for the allocation to one of four different conditions.

### Participants

Participants were recruited by written and online flyers that were distributed from March to June 2017 at Leiden University. Inclusion criteria were: (1) being fluent in Dutch and (2) being between 18 and 35 years old. Exclusion criteria were: (a) severe somatic or psychiatric conditions (e.g., chronic somatic diseases that affect daily life or Diagnostic and Statistical Manual of Mental Disorders-Fourth Edition Text Revision [DSM-IV-TR] psychiatric disorders) interfering with the study protocol, (b) current or recent (< 3 months ago) stressful life events interfering with daily life, and/or (c) heavy drug or alcohol (≥ 3 units a day) use.

### Experimental conditions and control condition

In both relaxation intervention conditions, i.e., with and without verbal suggestions, participants were provided with two brief relaxation exercises and participants were told that they would perform two relaxation exercises with a short break in between the exercises. Participants were instructed to sit upright in their chair with their feet on the ground and their arms resting on their thighs or on the seat rests. Subsequently, the experimenter provided the participant with Sennheiser HD201headphones and instructed them to start the audio file when they were ready to listen to the relaxation exercises. The experimenter then left the room. The first relaxation exercise focused on progressive muscle relaxation and led participants through a series of practices to tense and relax various muscle groups throughout the whole body. This relaxation exercise took around 16 minutes. Thirty seconds after the end of the first exercise, the second visual imagery-based relaxation exercise automatically started, which was focused on visualization of optimal health. Participants had to visualize that they were feeling healthy and full of energy, and that their body was in an optimal condition. This intervention took around 5 minutes. Total duration of the relaxation intervention was 20–25 minutes.

The verbal suggestions focused on the effectiveness of the relaxation intervention in performing the follow-up challenge test with the aim to facilitate more positive outcome expectancies for this task and yielded some cues for better performance on this task. More specifically, the experimenter provided participants with the following verbal suggestions (translated from Dutch):

*“In a moment, you will complete some tasks, including an arithmetic task. Prior research has shown that you can complete those tasks better when you relax by*:

1. Sitting as restful and relaxed as possible;
2. *Keeping in mind that your respiration has to be restful and regular, calm and relaxed*.

It is important that you keep this information in mind. You need this information in order to perform well on the tasks. Therefore, be careful not to forget these instructions.”

In the brief relaxation intervention plus verbal suggestions condition, participants were first provided with the verbal suggestions followed by the relaxation intervention. In the verbal suggestions only condition, participants did not receive the relaxation exercises, but were exposed to the stress task directly after receiving the verbal suggestions.

In the control condition, participants completed several neutral word finding puzzles for 25 minutes before they were exposed to the TSST.

### Psychosocial stress challenge

In order to provide participants with a psychosocial stress challenge, participants were subjected to the TSST [16]. This validated and commonly used task contains a mock job interview and a mental arithmetic task, which have to be performed in front of a two-member panel of judges. In order to induce anticipatory stress, participants were given 5 minutes to prepare a presentation about their preferred future job position. Subsequently, participants had to present themselves in front of a two-member panel of judges. During the presentation, the panel members pretended to take notes and asked some critical questions without providing any personal feedback to the participants. Also, participants were informed that the presentation was recorded with a video-camera and a voice recorder. After 6 minutes, participants were instructed to count backwards in steps of 17 from 1965 to 0. When participants provided the wrong answer or did not answer fast enough, they were told to start over again from 1965. After 2 minutes, all participants were told that they made too many mistakes or that they responded too slowly and that they would be provided with an ‘easier’ task (count backwards in steps of 13 from 1687 to 0), which actually reflected the same level of difficulty. The duration of this mental arithmetic part was 4 minutes and the total TSST procedure took around 15 minutes. This task has been found to reliably induce psychological, neuroendocrine, and autonomic nervous system responses [17, 18].

### Self-reported outcomes

To control for chronic stress, we included the Perceived Stress Scale (PSS) [19]. Participants completed 10 items concerning feelings and thought of the 4 last weeks on a 5-point scale ranging from 1 (*never*) to 5 (*very often*), with higher scores representing higher perceived stress. The PSS was found to have a good internal reliability in previous literature (Cronbach’s alpha = .85) [20], as well as in the present study (Cronbach’s alpha = .83).

#### State anxiety

State anxiety was measured by the short state version of the State Trait Anxiety Inventory (STAI-S-s) [21]. Participants completed 6 items on a 4-point scale ranging from 1 (*not at all*) to 4 (*very much*), such as ‘I am tense’. Scores on this scale can range from 6 to 24, with higher scores representing higher state anxiety. The Dutch translation was found to have a good internal reliability in previous literature (Cronbach’s alpha = .90) [22], as well as in the present study (Cronbach’s alpha = .78).

#### Well-being

Self-reported well-being was measured by a Numeric Rating Scale (NRS) [23]. Participants completed 7 items on an 11-point rating scale ranging from 0 (*not at all*) to 10 (*very much*), such as ‘How calm do you feel at this moment?’. Total scores on this scale can range from 0 to 70, with higher scores representing higher well-being. The NRS was found to have a good internal reliability in the present study (Cronbach’s alpha = .86).

#### Positive and negative affect

In addition, a Dutch version of the Positive and Negative Affect Schedule (PANAS) was used in order to measure affect [24]. Participants completed 20 items on a 5-point scale ranging from 1 (*barely or totally not*) to 5 (*very*), such as ‘inspired’ or ‘nervous’. For each scale, scores can range from 10 to 50 with higher scores on the positive affect scale indicating higher self-reported positive affect and higher scores on the negative affect scale indicating higher negative affect. The PANAS was found to have a good internal reliability in previous literature (Cronbach’s alpha positive affect scale = .88; Cronbach’s alpha negative affect scale = .87) [25], as well as in the present study (Cronbach’s alpha positive affect scale = .85; Cronbach’s alpha negative affect scale = .72).

### Psychophysiological outcomes

#### Cortisol and alpha-amylase

Saliva samples were collected with Sarstedt salivettes (Sarstedt, Germany) in order to measure cortisol and alpha-amylase. Saliva was separated from the cotton swab by centrifugation and stored at -80°C until analyzed. Cortisol was determined with a competitive electrochemiluminescence immunoassay ECLIA using a Modular Analytics E602 immunoassay analyzer on the Roche Cobas 8000 from Roche Diagnostics (Mannheim, Germany). Determination of salivary alpha-amylase was performed using a colorimetric method with Ethylidene Protected Substrate (EPS) on the Roche Cobas 8000 C702 and C502 (Mannheim, Germany).

#### Heart rate and skin conductance

Heart rate and skin conductance levels were measured continuously with a BIOPAC MP150 system using Acknowledge software version 4.4.1. The electrocardiogram (ECG) signal was recorded with an ECG100C module set at 1000Hz. The gain was set at 1000, the high pass filter was set at 0.05Hz and the low pass filter was set at 35Hz. For skin conductance level, Ag/Agcl electrodes were attached at the medial phalange of the index and middle finger of the non-dominant hand. Skin conductance was measured using a GSR100C module set at 1000Hz with a gain of 5μƱ/V and a low pass filter at 10Hz. The Physio Data Toolbox Version 0.1 [26] was used for visual inspection of the data as well as for calculating the mean heart rate and skin conductance levels for each of the specific time points. Due to technical problems, data of skin conductance were not reliable for 4 participants and a substantial number of artifacts (e.g., extra systoles, frequent movements during the measurements) in the heart rate data was found for 5 participants, resulting in exclusion of those data from further data analyses.

### Procedure

Prior to participation, participants were informed that the experiment was about attention processes and arithmetic skills, without providing further information on the actual study purpose. Participants provided written informed consent. Next, several online questionnaires probing the eligibility criteria, demographics, and participant characteristics not related to the present study aim were completed. If participants were eligible to participate in the study, they were invited for a single lab session that took place at the Faculty of Social and Behavioural Sciences of Leiden University, the Netherlands. Participants were instructed to refrain from using alcohol and drugs 24 hours before the lab session, to refrain from heavy physical activity starting from the evening before the lab session, and to refrain from consuming caffeine, smoking, and consuming warm meals 2 hours before the lab session. All lab sessions were planned in the afternoon, in order to limit the influence of the cortisol awakening response on the neuroendocrine measures.

At the start of the lab session, baseline self-reported perceived stress, state anxiety, well-being, and positive and negative affect were assessed. Thereafter, participants were connected to the equipment during the whole lab session, in order to measure resting state heart rate and skin conductance continuously. In addition, heart rate and skin conductance were also measured in resting state for 5 minutes at 5 different time points: baseline, after the intervention, directly after the TSST, and 10 and 20 minutes after the TSST. For all those measurements, participants were instructed to sit quietly and to move as little as possible. For the saliva sampling, participants were asked to hold the cotton swab in their mouth for one minute and to move it around without chewing on it, since less saliva will remain in the cotton swab after chewing on it. Thereafter, participants were randomized to one of four conditions and received the relaxation or control exercises and/or verbal suggestion, followed by completion of the questionnaires and psychophysiological measures (2^nd^ measurement). Subsequently, participants were exposed to the TSST. Directly after the TSST, as well as 10 and 20 minutes afterwards, participants again completed the questionnaires and the psychophysiological measures (3^rd^, 4^th^, and 5^th^ measurement). At the end of the session, participants were debriefed about the actual study purpose and were requested not to provide information about the actual study aim to future participants. The total study duration was about 2 hours and participants received compensation for their participation (€15 or course credits).

### Data preparation and statistical analyses

Based on a previous study on the effectiveness of a brief stress management intervention on state anxiety, an effect size around *f* = .27 was expected [9]. A sample size of 30 participants in each condition (total 120 participants) allowed sufficient statistical power (power = .80) to detect this effect size with α = .05, as calculated by G*power 3.1 [27].

IBM SPSS Statistics for Windows (Version 23; IBM Corporation, Armonk, NY, USA) was used to analyze the data with a two-tailed significance level (α = .05). To test the primary hypothesis that a brief relaxation intervention, with or without verbal suggestions, would result in decreased self-reported state anxiety, mixed repeated measures analyses of variance (RM ANOVAs) were conducted with the within-subjects factor Time (i.e., 5 levels: baseline, post-intervention, and post-, 10 min, and 20 min after TSST) and the between-subjects factor Type of Manipulation (both relaxation intervention conditions vs. control) [28]. A check on the stressfulness of the TSST was performed by examining the significance of the factor Time was performed before the primary outcome of interest, i.e., the interaction effect of Time and Type of Manipulation. To examine at which time point(s) groups differed on state anxiety when a significant interaction effect was found between Time and Type of Manipulation, Holm’s corrected ANOVAs were performed to compare both relaxation intervention conditions with the control condition at specific time intervals by calculating difference scores between baseline and each of the other time points. Next, to further investigate the role of verbal suggestions, Holm’s corrected pairwise comparisons were performed on the time intervals that indicated significant differences to compare all four conditions separately. To investigate the effects of the relaxation intervention, with or without verbal suggestions, for the secondary self-reported outcomes well-being and positive and negative affect, analyses were performed in a similar way as for self-reported state anxiety. For heart rate and skin conductance, the analyses were performed in a similar way as described above, although the within-subjects factor Time contained 7 time points instead of 5 (i.e., baseline, during manipulation, after manipulation, during TSST, after TSST, 10 min after TSST, and 20 min after TSST). For cortisol and alpha-amylase, the area under the curve was calculated with respect to the ground (AUC_g_), irrespective of the time distance between measurement points [29]. To test the effects on AUC_g_ cortisol and alpha-amylase, between-subjects ANOVAs (with the between-subjects factor Type of Manipulation) and subsequent pairwise comparisons were performed in a similar way as described above (without the within-subjects factor Time).

## Results

### Participant characteristics

**Fig 1:**
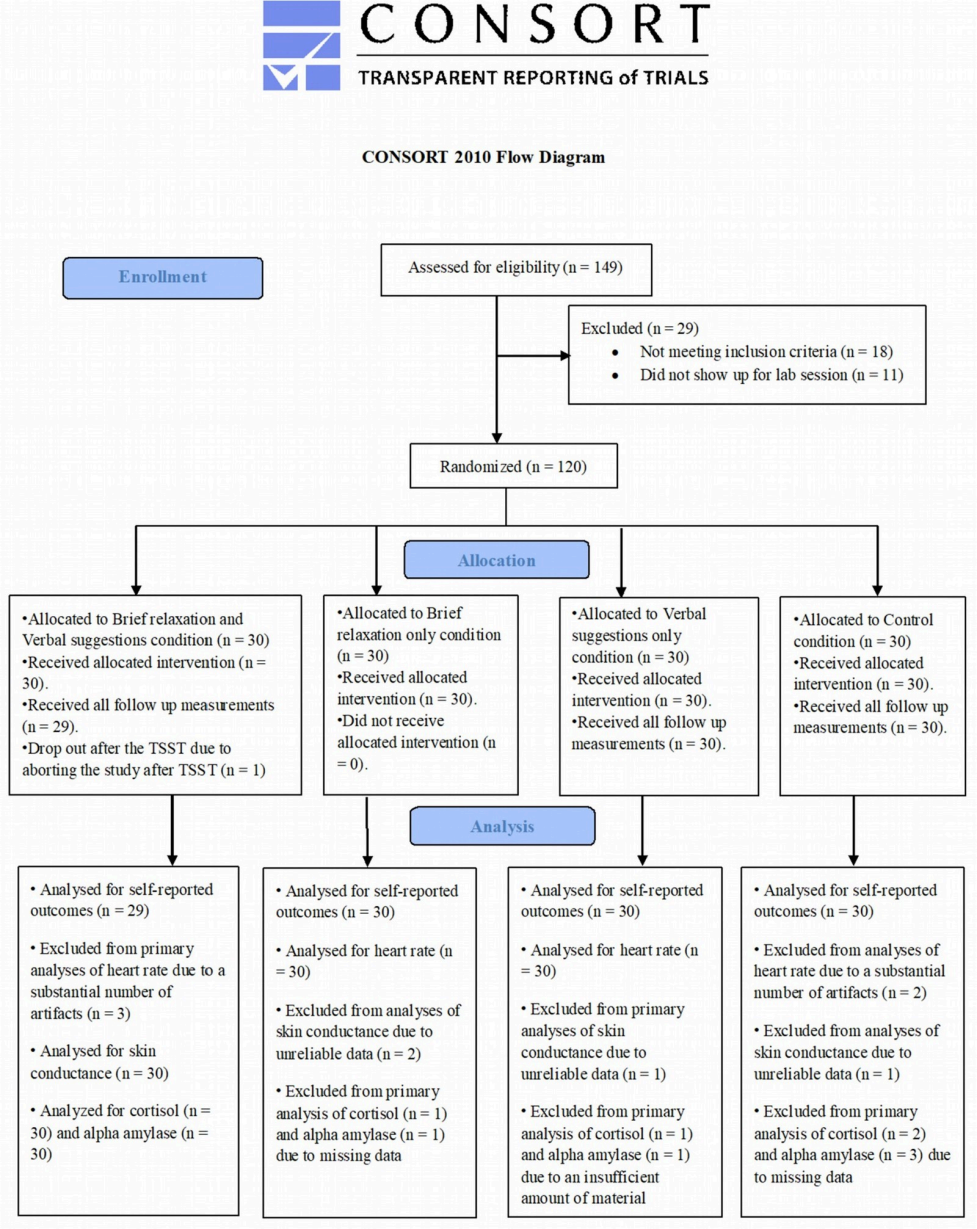
Flowchart of the present study.

See Fig 1 for the flowchart of the present study. Of the 149 participants who completed the online questionnaire, 131 were found eligible to participate in the present study. Eleven participants did not show up for the planned lab session, resulting in 120 participants (97 women; 80.8%) with a mean age of 22.1 years (*SD* = 2.3; range = 18–29) who completed the present study (30 in each condition). One participant (in the brief relaxation plus verbal suggestions condition) aborted the study after completion of the TSST and therefore, data of this participant are only available until completion of the TSST. Baseline characteristics of all participants are presented in Table 1. No significant group differences were found on demographics and baseline levels of the self-reported and psychophysiological outcome measures, as indicated by separate ANOVAs (all *p* > .05).

**Table 1:** Descriptive baseline statistics (mean and standard deviations) for the four conditions separately.

	Brief relaxation intervention + verbal suggestions (*N* = 30)	Brief relaxation intervention only (*N* = 30)	Verbal suggestions only (*N* = 30)	Control (*N* = 30)
Sex, *n* female (%) *Mean (SD)*	26 (86.7%)	23 (76.7%)	23 (76.7%)	25 (83.3%)
Age	22.5 (2.1)	22.0 (2.4)	21.7 (2.3)	22.3 (2.2)
Perceived stress	22.8 (4.2)	22.7 (5.6)	23.7 (4.5)	23.1 (5.8)
State anxiety	8.9 (2.1)	9.3 (3.0)	10.1 (2.7)	9.6 (2.4)
Well-being	56.7 (6.4)	55.9 (9.1)	53.7 (8.6)	54.3 (8.4)
Positive affect	28.4 (6.9)	27.0 (6.1)	26.8 (7.2)	27.6 (6.5)
Negative affect	12.2 (2.5)	12.0 (2.7)	12.4 (2.6)	11.9 (2.3)
Heart rate in beats per minute	74 (10)^1^	70 (8)	74 (9)	73 (8)^2^
Skin conductance in microsiemens	3.5 (1.9)^3^	2.6 (1.0)^1^	3.7 (1.7)^3^	3.3 (1.5)
Cortisol in nmol/L	5.6 (3.1)	4.5 (1.8)^3^	6.3 (2.1)^3^	5.1 (3.0)^3^
Alpha-amylase in U/L	103.4 (66.5)	140.0 (93.8)^3^	117.3 (98.4)^3^	172.1 (126.9)^3^

**Note**. *SD* = standard deviation, nmol/L = nanomoles/liter, U/L = units/liter.

^1^*N* = 28

^2^*N* = 27

^3^*N* = 29

### State anxiety

**Fig 2:**
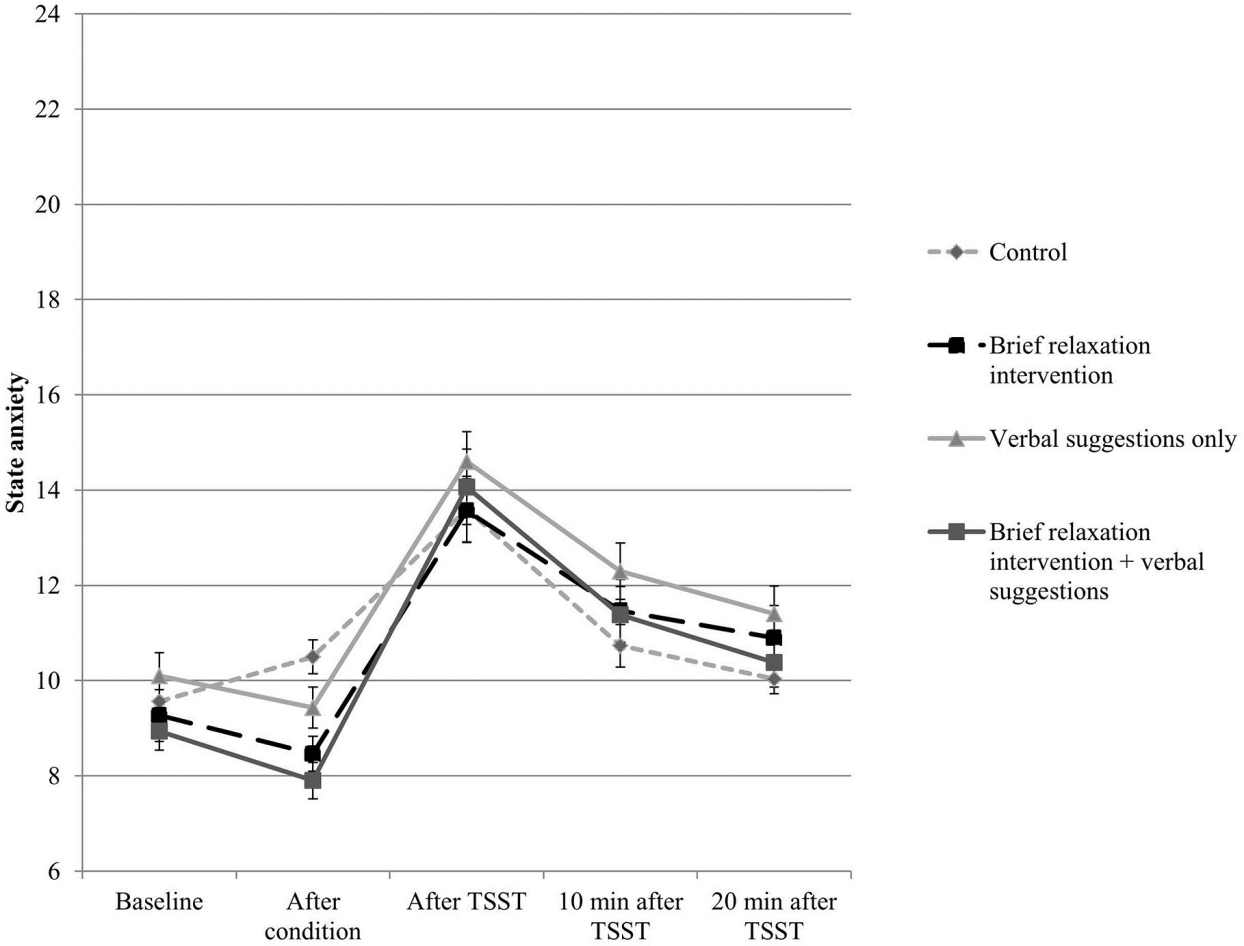
State anxiety levels for the four conditions on the various time points. The x-axis represents the various time points, whereas the y-axis represents the level of self-reported state anxiety. A higher score on the y-axis represents a higher level of state anxiety.

The results for self-reported state anxiety are presented in Fig 2. A significant main effect of Time was found (*F*(2.76, 240.12) = 63.63, *p* < .001, *n*^*2*^ = .42), indicating that the TSST was effective in increasing self-reported state anxiety (see Fig 2). In addition, we observed a significant interaction effect between Time and Type of Manipulation (*F*(2.76, 240.12) = 7.65, *p* < .001, *n*^*2*^ = .08), indicating lower self-reported state anxiety after the condition for both intervention conditions combined (with and without verbal suggestions) compared to the control condition (see Fig 2). Holm’s corrected ANOVAs showed a significant difference between the combined intervention groups and the control condition post-intervention (*F*(1, 88) = 10.56, *p adjusted* < .01, *n*^*2*^ = .11). When investigating all four groups on this time interval, Holm’s corrected pairwise comparisons showed significantly smaller increase in state anxiety from baseline to post-intervention state anxiety in both the brief relaxation intervention plus verbal suggestions condition (*M* = -1.03, *SD* = 2.62; *t*(58) = 3.07, *p adjusted* = .018) and the brief relaxation intervention only condition (*M* = -0.80, *SD* = 2.71; *t*(58) = 2.66, *p adjusted* = .050) as compared to the control condition (*M* = 0.93, *SD* = 2.33). No significant differences were found for the other pairwise comparisons.

### Well-being

**Fig 3:**
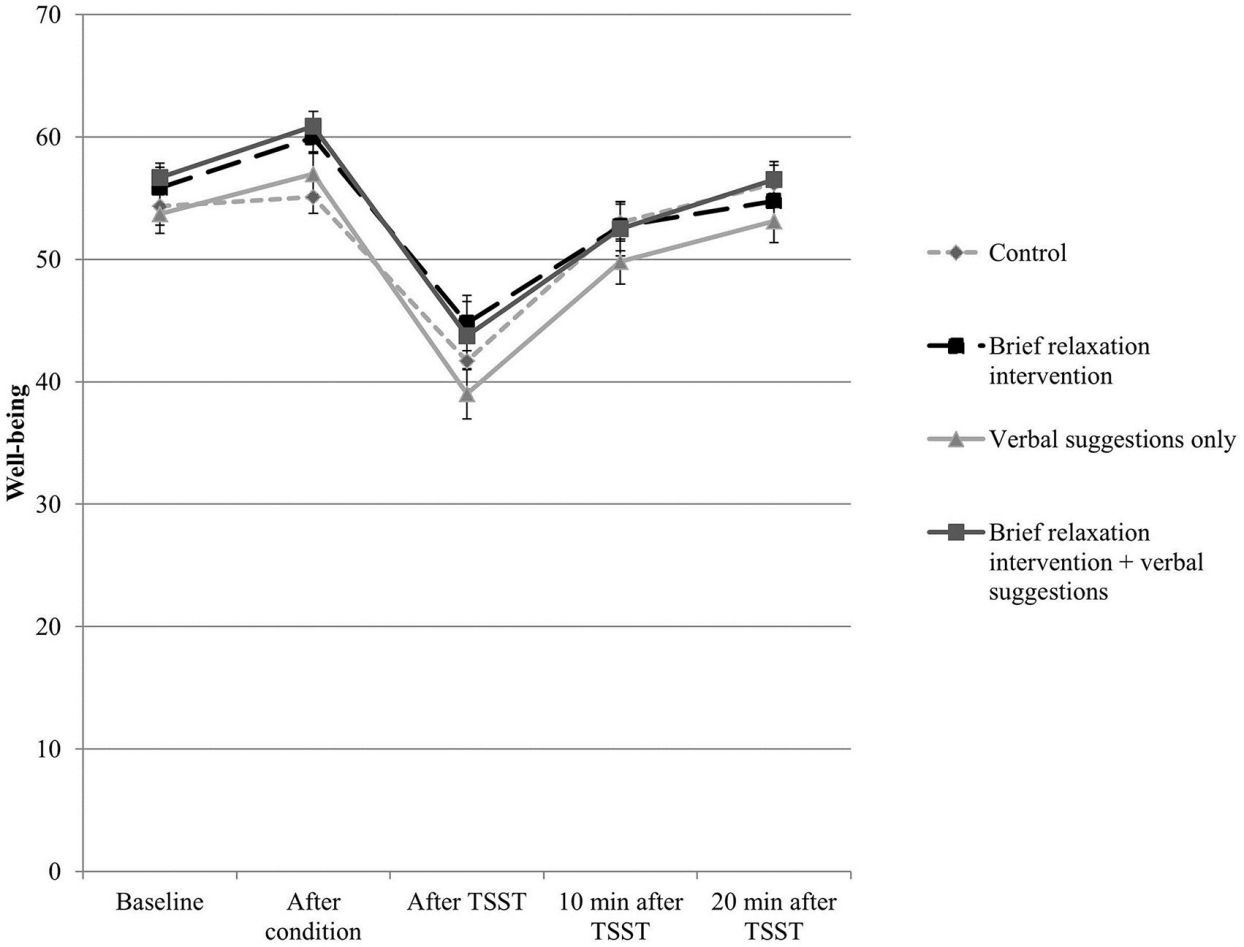
Levels of well-being for the four conditions on the various time points. The x-axis represents the various time points, whereas the y-axis represents the level of self-reported well-being. A higher score on the y-axis represents a higher level of well-being.

The results for well-being are presented in Fig 3. A significant main effect of Time was found (*F*(2.11, 183.34) = 62.81, *p* < .001, *n*^*2*^ = .42), indicating that the stress manipulation was effective in reducing self-reported well-being (see Fig 3). A trend towards significance was found for the interaction effect between Time and Type of Manipulation (*F*(2.11, 183.34) = 2.92, *p* = .05, *n*^*2*^ = .03), indicating higher self-reported well-being after the condition for both intervention conditions combined (with and without verbal suggestions) compared to the control condition (see Fig 3). Exploratory pairwise comparisons did not yield any significant group differences.

### Positive and negative affect

**Fig 4:**
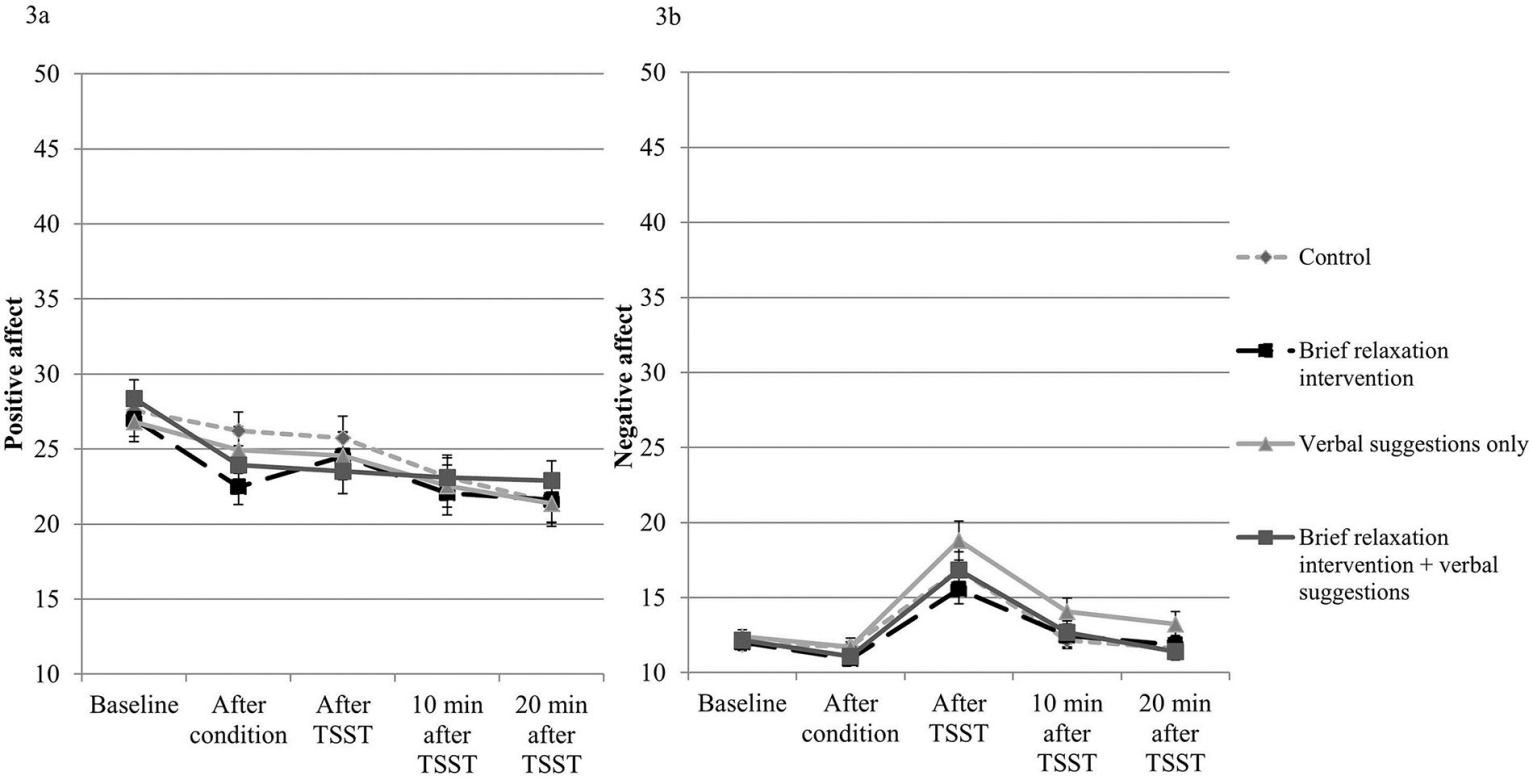
Levels of positive affect (Fig 4a) and negative affect (Fig 4b) for the four conditions on the various time points. The x-axis represents the various time points, whereas the y-axis represents the level of self-reported positive affect or negative affect, respectively. A higher score on the y-axis represents a higher level of positive affect or negative affect, respectively.

The results for positive and negative affect are presented in Fig 4a and 4b, respectively. For positive affect, a significant main effect of Time was found (*F*(3.35, 291.70) = 19.75, *p* < .001, *n*^*2*^ = .19), indicating that the TSST reduced self-reported positive affect. No significant interaction effect was found between Time and Type of Manipulation, indicating that positive affect was not influenced differentially between conditions.

For negative affect, a significant main effect of Time was found, *F*(1.83, 158.96) = 39.40, *p* < .001, *n*^*2*^ = .31, indicating that the TSST was effective in increasing self-reported negative affect. No interaction effect was found between time and Type of Manipulation, indicating that negative affect was not influenced differentially between conditions.

### Heart rate and skin conductance

**Fig 5:**
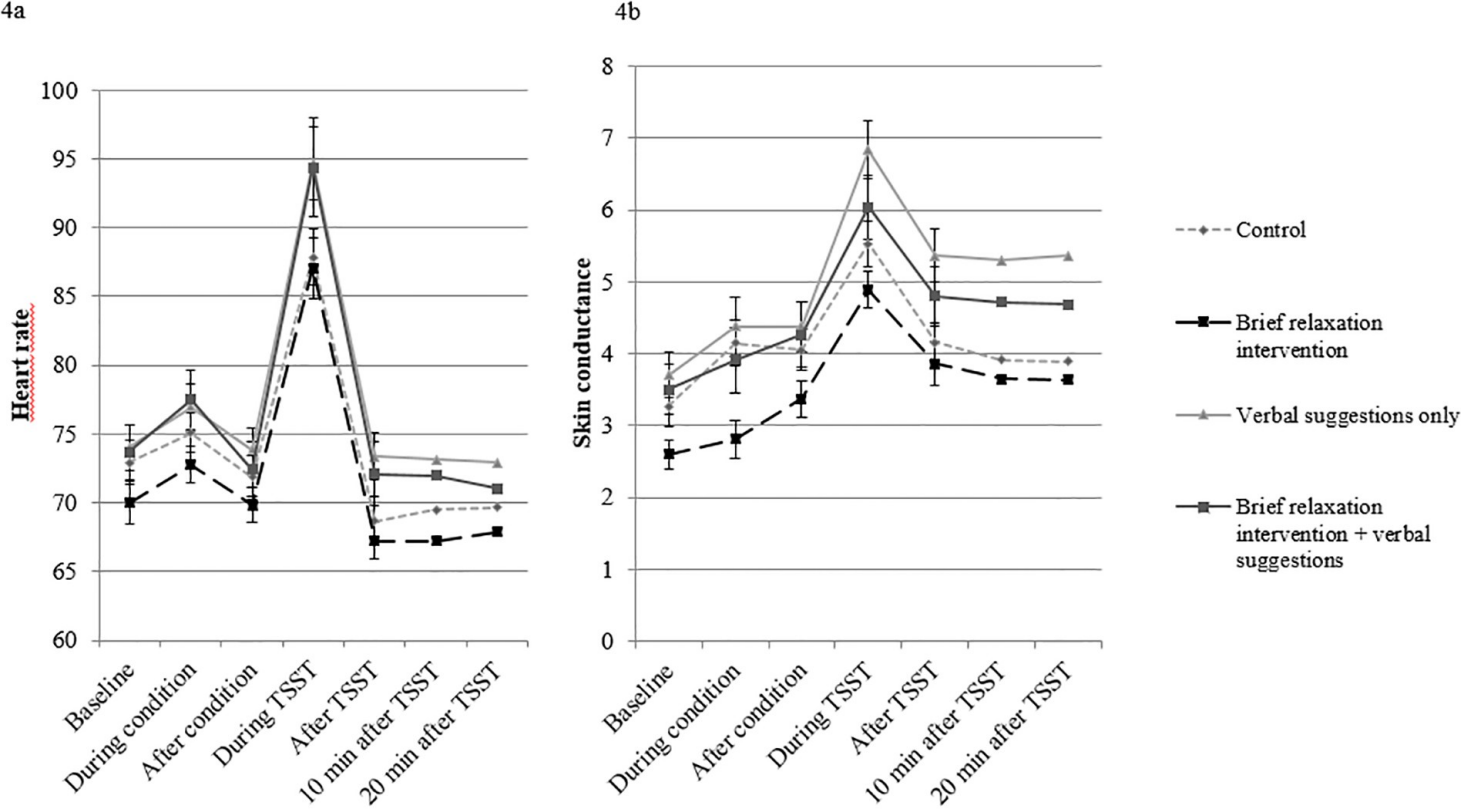
Mean heart rate in beats per minute (Fig 5a) and skin conductance in microsiemens (Fig 5b) for the four conditions on the various time points during the lab session. The x-axis represents the various time points, whereas the y-axis represents the mean heart rate and skin conductance, respectively. A higher unit on the y-axis represents a higher mean heart rate level or skin conductance, respectively. Note that the results are presented in a restricted range for a more clear view on the data.

The results for heart rate and skin conductance are presented in Fig 5a and 5b, respectively. For heart rate, a significant main effect of Time was found (*F*(1.85, 151.50) = 178.10, *p* < .001, *n*^*2*^ = .69), indicating that the TSST was effective in increasing heart rate. No significant interaction effect between Time and Type of Manipulation was observed, indicating that heart rate was not affected differentially between conditions.

For skin conductance, a significant main effect of Time was found (*F*(2.43, 203.70) = 68.68, *p* < .001, *n*^*2*^ = .45), indicating that the TSST was effective in increasing skin conductance. In addition, a significant interaction effect between Time and Type of Manipulation was observed (*F*(2.43, 203.70) = 4.67, *p* = .007, *n*^*2*^ = .05), showing that skin conductance was affected differentially between conditions. Holm’s corrected ANOVAs showed no significant differences between the combined relaxation intervention groups and the control group on any of the specific time intervals. Exploratory, pairwise comparisons across time points showed a significant difference between the control condition and verbal suggestions only condition (*F* (2.20, 125.49) = 8.61, *p adjusted* < .001, *n*^*2*^ = .13), with no other significant group differences found (*p*-values > .05). Post hoc pairwise comparisons on the various time intervals showed a significantly higher skin conductance rise in the verbal suggestions versus control condition from baseline to during the TSST (*M* = 3.14, *SD* = 1.10 vs. *M* = 2.27, *SD* = 1.02; *p adjusted* = .01), directly post- TSST (*M* = 1.66, *SD* = 1.31 vs. *M* = 0.89, *SD* = .99; *p adjusted* = .03), 10 minutes after the TSST (*M* = 1.60, *SD* = 1.47 vs. *M* = 0.65, *SD* = .97; *p adjusted* = .02), and 20 minutes after the TSST (*M* = 1.66, *SD* = 1.43 vs. *M* = .62, *SD* = .93; *p adjusted* = .01).

### Cortisol and alpha-amylase

**Fig 6:**
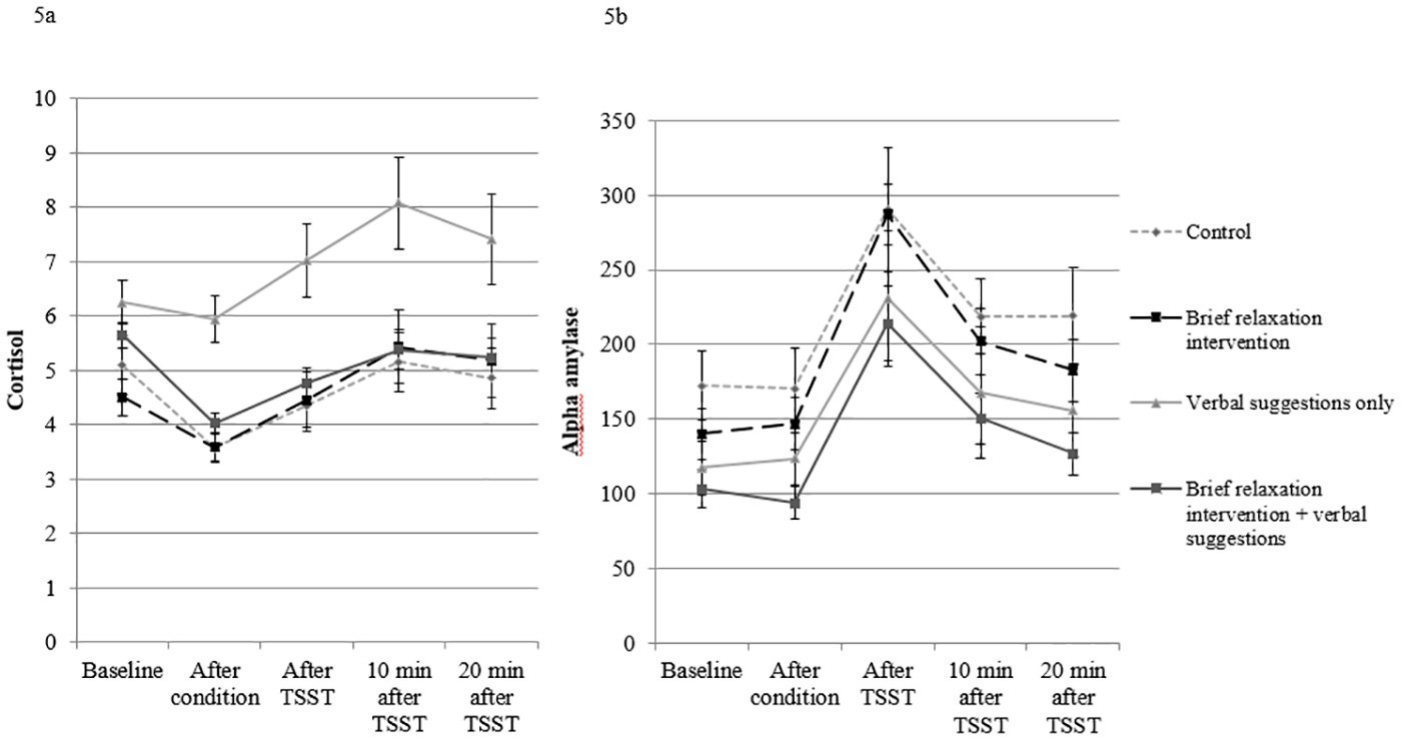
Levels of cortisol in nanomoles per liter (Fig 6a) and alpha-amylase in units per liter (Fig 6b) for the four conditions on the various time points during the lab session. The x-axis represents the various time points, whereas the y-axis represents the level of cortisol and alpha-amylase, respectively. A higher unit on the y-axis represents a higher level of cortisol or alpha-amylase, respectively. Note that the results are presented in a restricted range for a more clear view on the data.

Fig 6a and 6b present the results on cortisol and alpha-amylase, respectively. For cortisol, the AUC_g_ data were not normally distributed and, therefore, a logarithmic transformation was applied. No significant group differences were found for cortisol (*p* > .60).

For the AUC_g_ data on alpha-amylase, a trend towards significance was found (*F* (1, 84) = 3.44, *p* = .07), in that both intervention conditions combined showed a marginally lower level of AUC_g_ alpha-amylase compared to the control condition.

## Discussion

The present experimental proof-of-concept study investigated for the first time the effects of a brief relaxation intervention and the role of verbal suggestions on stress-related outcomes by exposing participants between 18 and 35 years of age to a psychosocial stress challenge. After the brief relaxation interventions (with or without verbal suggestions), lower self-reported state anxiety was found compared to the control condition. This effect was only seen directly after relaxation, however, and did not impact the subsequent psychosocial stress challenge. No significant effects of the relaxation interventions were found for other self-reported outcomes or the psychophysiological outcome data. In addition, no support was found for the add-on effectiveness of verbal suggestions. By applying an innovative design including four different conditions and evaluating them with various self-reported as well as psychophysiological outcome measures, the present study provides preliminary support for the effectiveness of a brief relaxation intervention on reducing state anxiety.

The present study demonstrated the effectiveness of a brief relaxation intervention in reducing state anxiety and, moreover, was the first in investigating whether verbal suggestions, based on inducing positive outcome expectancies, can strengthen the effects of a relaxation intervention. This study did not find support for the add-on effectiveness of verbal suggestions. Nonetheless, it is premature to conclude that verbal suggestions do not have any add-on effects on relaxation interventions. As the present experimental proof-of-concept study first evaluated the effectiveness of relaxation on the stress response, before evaluating the add-on effectiveness of the verbal suggestions, the present study had a good power to detect the effects of both brief relaxation interventions versus the control condition, but potentially lower power to detect effects in the separate conditions. Therefore, more research with larger sample sizes is needed to gain more insight into the specific add-on effects of the verbal suggestions. When determining the required sample size, future studies should take the effect size of the verbal suggestions only condition or the combined relaxation and verbal suggestions condition into account, depending on their specific research question. In the placebo literature, the effectiveness of verbal suggestions on health outcomes is well-described [13, 14]. Moreover, the verbal suggestions used in the present study were formulated rather generic (i.e., to address a broad target population) encompassing multiple relaxation components, whereas verbal suggestions in placebo literature are usually focused on a specific sensation or manipulation [30]. It could be that participants did not benefit from the verbal suggestions just because they could not pick up the link between the verbal suggestions and the upcoming stress challenge. Future studies should therefore investigate whether formulating the verbal suggestions more specifically (e.g., providing concrete examples of the benefits of stress management and providing more information on the link between the components of the verbal suggestions and the actual relaxation response) can optimize stress responses. In addition, the effectiveness of the verbal suggestions should be further elucidated in future research by incorporating various types of verbal suggestions, e.g., varying in the details that are provided concerning stress-management techniques, the details of the effectiveness of stress management, as well as varying the number of times the verbal suggestions are provided to participants or by providing participants with booster verbal suggestions (e.g., by exposing participants in the verbal suggestions only condition with multiple booster suggestions, as in the present study this condition was provided only once with the verbal suggestion and directly followed by the psychosocial stress challenge). The role of verbal suggestions in optimizing psychological interventions is not only of scientific relevance, but also of clinical relevance. When it turns out that verbal suggestions are an effective add-on tool, the communication between patients and health professionals can be optimized by applying verbal suggestions to influence outcome expectancies [31]. Due to ethical concerns, an open-label approach seems to be the most appropriate approach to use in communication with patients [32].

The brief relaxation intervention resulted in lower self-reported state anxiety after both brief relaxation intervention conditions combined (with and without verbal suggestions) compared to the control condition. However, no group differences were observed after the psychosocial challenge. This finding is in accordance with a previous pilot study, also reporting a significantly lower self-reported state anxiety after a stress management intervention, but not after the TSST [9]. The results for self-reported well-being were in line with the results for self-reported state anxiety as a trend was found for a higher level of self-reported well-being after both brief relaxation interventions combined (with and without verbal suggestions), compared to the control condition, although pairwise comparisons did not yield any significant differences. As the results on state anxiety were only seen directly after the intervention and not in response to the psychosocial stress challenge, it might be that a brief relaxation intervention is not effective enough to buffer the effects of stress in response to a challenge. An alternative explanation can be found in the type of stressor that was used. The TSST is a well-validated stressor that is commonly used in research [17]. It is, however, a rather robust acute stressor and as all participants show increased stress levels by this task, it might therefore be difficult to differentiate between the stress responses of the present incorporated experimental and control conditions after the TSST. A previous study comparing the TSST to another stressor, i.e., a cold pressor test (CPT), demonstrated that the TSST was most effective in activating the hypothalamic-pituitary-adrenal (HPA) and sympathetic-adrenal-medullary (SAM) axis, whereas with the CPT a differential response pattern between various included populations could be identified. However, the CPT concerns a stressor of shorter duration, which can impact the time course of the response [33]. Future research might therefore incorporate various stressors (e.g., anticipatory and social evaluative stressors as well as a more physical stressors) in order to investigate the potential generalizing effects of a brief relaxation intervention to other stressors and the potential added value of verbal suggestions thereon. In addition, future studies might consider incorporating participants who are at risk for inadequate coping with stress (e.g., participants with high trait anxiety), as well as participants with a predisposition for high levels of stress in order to see whether the intervention can be used to optimize stress management skills in specific target populations.

For the psychophysiological outcomes, a trend was found for both brief relaxation intervention conditions combined (with and without verbal suggestions) showing a lower overall alpha-amylase concentration as compared to the control condition. As lower concentrations of alpha-amylase are related to lower autonomic nervous system arousal, this finding is in accordance with our expectation that both brief relaxation intervention conditions would result in a lower stress response. However, this finding should be interpreted with caution, since only a trend was found. Additionally, this trend was not supported by the other psychophysiological outcome measures, as no significant group differences were found for heart rate (also reflecting autonomous nervous system reactivity) and cortisol (reflecting activation of the HPA-axis). The results on the psychophysiological outcome data in the present study, therefore, warrant further research.

Next to the innovative features incorporated in the present study, i.e., incorporating four different experimental conditions, incorporating a psychosocial challenge to observe the effects of a brief relaxation intervention accompanied or not with a verbal suggestion on state anxiety, and incorporating self-reported as well as psychophysiological outcome measures, there are some limitations that should be noted as well. First of all, the present study was based on a rather highly educated sample, limiting its generalizability. Second, although TSST panel members and test leaders had to behave strictly according to a predefined script and we do not have indications that their behaviors varied along the condition to which participants were randomized, research personnel were not blinded to the allocation procedures. In order to exclude any performance bias, future studies should make both test leaders and TSST panel members blind for allocation by including a third independent researcher that performs randomization and subsequently completes the condition with participants, but is not involved in other parts of the study. Third, we did not include a manipulation check to evaluate whether the verbal suggestions were credible to participants. Finally, the present study did incorporate a brief relaxation intervention that was not necessarily tailored to the specific needs of the participant. Possibly, some participants already possessed the skills that were provided to them in the brief relaxation intervention, whereas others might have had difficulties with acquiring coping skills from the intervention. Therefore, future studies may tailor the intervention by guiding the relaxation practice according to the specific challenge they are faced with and evaluate whether participants actually pick up the link between the instructions and handling with the upcoming stress challenge.

In conclusion, the present experimental proof-of-concept study found some preliminary support for the effectiveness of a brief relaxation intervention, with or without verbal suggestions, in decreasing state anxiety. These effects occurred directly after the condition, but not after a psychosocial challenge. No add-on effect of verbal suggestions was found in the present study. Future studies should further investigate the effectiveness of brief relaxation interventions on stress responses by tailoring the intervention to the specific challenges and by incorporating other (more sensitive) psychosocial challenges. In addition, future research should further elucidate the role of verbal suggestions by incorporating various types of verbal suggestions and manipulate the number of times the verbal suggestions are provided.

## Supporting information

S1 Text Consort checklist. (DOC)

S2 Text Protocol, Dutch version. (PDF)

S3 Text Protocol, English version. (PDF)

## References

[1] Cohen S, Hamrick N, Rodriguez MS, Feldman PJ, Rabin BS, Manuck SB. Reactivity and vulnerability to stress-associated risk for upper respiratory illness. Psychosomatic medicine. 2002;64(2):302–10. Epub 2002/03/27. . 11914447 10.1097/00006842-200203000-00014

[2] Segerstrom SC, Miller GE. Psychological stress and the human immune system: a meta-analytic study of 30 years of inquiry. Psychological bulletin. 2004;130(4):601–30. Epub 2004/07/15. 10.1037/0033-2909.130.4.601 . 15250815 PMC1361287

[3] Sharma M, Rush SE. Mindfulness-based stress reduction as a stress management intervention for healthy individuals: a systematic review. Journal of evidence-based complementary & alternative medicine. 2014;19(4):271–86. Epub 2014/07/24. 10.1177/2156587214543143 . 25053754

[4] Richardson KM, Rothstein HR. Effects of occupational stress management intervention programs: a meta-analysis. Journal of occupational health psychology. 2008;13(1):69–93. Epub 2008/01/24. 10.1037/1076-8998.13.1.69 . 18211170

[5] van Dixhoorn JJ, Duivenvoorden HJ. Effect of relaxation therapy on cardiac events after myocardial infarction: a 5-year follow-up study. Journal of Cardiopulmonary Rehabilitation and Prevention. 1999;19(3):178–85. 10.1097/00008483-199905000-00005 10361649

[6] Nagele E, Jeitler K, Horvath K, Semlitsch T, Posch N, Herrmann KH, et al Clinical effectiveness of stress-reduction techniques in patients with hypertension: systematic review and meta-analysis. Journal of hypertension. 2014;32(10):1936–44; discussion 44. Epub 2014/08/02. 10.1097/HJH.0000000000000298 . 25084308

[7] Creswell JD, Pacilio LE, Lindsay EK, Brown KW. Brief mindfulness meditation training alters psychological and neuroendocrine responses to social evaluative stress. Psychoneuroendocrinology. 2014;44:1–12. Epub 2014/04/29. 10.1016/j.psyneuen.2014.02.007 . 24767614

[8] Gaab J, Blattler N, Menzi T, Pabst B, Stoyer S, Ehlert U. Randomized controlled evaluation of the effects of cognitive-behavioral stress management on cortisol responses to acute stress in healthy subjects. Psychoneuroendocrinology. 2003;28(6):767–79. Epub 2003/06/19. . 12812863 10.1016/s0306-4530(02)00069-0

[9] Cruess DG, Finitsis DJ, Smith A-L, Goshe BM, Burnham K, Burbridge C, et al Brief stress management reduces acute distress and buffers physiological response to a social stress test. International Journal of Stress Management. 2015;22(3):270.

[10] Bartels DJ, van Laarhoven AI, van de Kerkhof PC, Evers AW. Placebo and nocebo effects on itch: effects, mechanisms, and predictors. European journal of pain (London, England). 2016;20(1):8–13. Epub 2015/09/30. 10.1002/ejp.750 . 26417885

[11] Rohrmann S, Hennig J, Netter P. Changing psychobiological stress reactions by manipulating cognitive processes. International journal of psychophysiology: official journal of the International Organization of Psychophysiology. 1999;33(2):149–61. Epub 1999/09/17. . 10489079 10.1016/s0167-8760(99)00036-7

[12] Rohrmann S, Hennig J, Netter P. Manipulation of physiological and emotional responses to stress in repressors and sensitizers. Psychology and Health. 2002;17(5):583–96.

[13] Colloca L, Miller FG. Role of expectations in health. Current opinion in psychiatry. 2011;24(2):149–55. Epub 2011/01/21. 10.1097/YCO.0b013e328343803b . 21248640

[14] Colloca L, Miller FG. How placebo responses are formed: a learning perspective. Philosophical transactions of the Royal Society of London Series B, Biological sciences. 2011;366(1572):1859–69. Epub 2011/05/18. 10.1098/rstb.2010.0398 . 21576143 PMC3130403

[15] Skvortsova A, Veldhuijzen DS, Van Middendorp H, Van den Bergh O, Evers AWM. Enhancing Placebo Effects in Somatic Symptoms Through Oxytocin. Psychosomatic medicine. 2018;80(4):353–60. Epub 2018/04/04 . 29613940 10.1097/PSY.0000000000000571 PMC5959206

[16] Kirschbaum C, Pirke KM, Hellhammer DH. The ‘Trier Social Stress Test’—a tool for investigating psychobiological stress responses in a laboratory setting. Neuropsychobiology. 1993;28(1–2):76–81. Epub 1993/01/01. 10.1159/000119004 . 8255414

[17] Allen AP, Kennedy PJ, Cryan JF, Dinan TG, Clarke G. Biological and psychological markers of stress in humans: focus on the Trier Social Stress Test. Neuroscience and biobehavioral reviews. 2014;38:94–124. Epub 2013/11/19. 10.1016/j.neubiorev.2013.11.005 . 24239854

[18] Yamakawa K, Matsunaga M, Isowa T, Kimura K, Kasugai K, Yoneda M, et al Transient responses of inflammatory cytokines in acute stress. Biological psychology. 2009;82(1):25–32. Epub 2009/05/19. 10.1016/j.biopsycho.2009.05.001 . 19446599

[19] Cohen S, Kamarck T, Mermelstein R. A global measure of perceived stress. Journal of health and social behavior. 1983:385–96. 6668417

[20] Cohen S. Perceived stress in a probability sample of the United States. 1988.

[21] Marteau TM, Bekker H. The development of a six-item short-form of the state scale of the Spielberger State-Trait Anxiety Inventory (STAI). The British journal of clinical psychology. 1992;31 (Pt 3):301–6. Epub 1992/09/01. . 1393159 10.1111/j.2044-8260.1992.tb00997.x

[22] Van der Ploeg H. De zelf-beoordelings vragenlijst (STAI-DY). Tijdschr Psychiatr. 1982;24:576–88.

[23] Hartrick CT, Kovan JP, Shapiro S. The numeric rating scale for clinical pain measurement: a ratio measure? Pain practice: the official journal of World Institute of Pain. 2003;3(4):310–6. Epub 2006/12/15. 10.1111/j.1530-7085.2003.03034.x . 17166126

[24] Watson D, Clark LA, Tellegen A. Development and validation of brief measures of positive and negative affect: the PANAS scales. Journal of personality and social psychology. 1988;54(6):1063 3397865 10.1037//0022-3514.54.6.1063

[25] Kercher K. Assessing Subjective Well-Being in the Old-Old:The PANAS as a Measure of Orthogonal Dimensions of Positive and Negative Affect. Research on Aging. 1992;14(2):131–68. 10.1177/0164027592142001

[26] Sjak-Shie EE. PhysioData Toolbox (Version 0.1). 2016.

[27] Faul F, Erdfelder E, Buchner A, Lang AG. Statistical power analyses using G*Power 3.1: tests for correlation and regression analyses. Behavior research methods. 2009;41(4):1149–60. Epub 2009/11/10. 10.3758/BRM.41.4.1149 . 19897823

[28] Schakel L, Veldhuijzen DS, Middendorp HV, Dessel PV, Houwer J, Bidarra R, et al The effects of a gamified approach avoidance training and verbal suggestions on food outcomes. PloS one. 2018;13(7):e0201309 Epub 2018/07/27. 10.1371/journal.pone.0201309 . 30048511 PMC6062074

[29] Pruessner JC, Kirschbaum C, Meinlschmid G, Hellhammer DH. Two formulas for computation of the area under the curve represent measures of total hormone concentration versus time-dependent change. Psychoneuroendocrinology. 2003;28(7):916–31. Epub 2003/08/02. . 12892658 10.1016/s0306-4530(02)00108-7

[30] Peerdeman KJ, van Laarhoven AI, Donders AR, Hopman MT, Peters ML, Evers AW. Inducing Expectations for Health: Effects of Verbal Suggestion and Imagery on Pain, Itch, and Fatigue as Indicators of Physical Sensitivity. PloS one. 2015;10(10):e0139563 Epub 2015/10/09. 10.1371/journal.pone.0139563 . 26448183 PMC4598027

[31] Evers AWM, Colloca L, Blease C, Annoni M, Atlas LY, Benedetti F, et al Implications of Placebo and Nocebo Effects for Clinical Practice: Expert Consensus. Psychotherapy and psychosomatics. 2018;87(4):204–10. Epub 2018/06/13. 10.1159/000490354 . 29895014 PMC6191882

[32] Blease C, Colloca L, Kaptchuk TJ. Are open-Label Placebos Ethical? Informed Consent and Ethical Equivocations. Bioethics. 2016;30(6):407–14. Epub 2016/02/04. 10.1111/bioe.12245 . 26840547 PMC4893896

[33] McRae AL, Saladin ME, Brady KT, Upadhyaya H, Back SE, Timmerman MA. Stress reactivity: biological and subjective responses to the cold pressor and Trier Social stressors. Human psychopharmacology. 2006;21(6):377–85. Epub 2006/08/18. 10.1002/hup.778 . 16915579

