# Supplementary material for: Can verbal suggestions strengthen the effects of a relaxation intervention?
Source: PLoS One. 2019 Aug 7;14(8):e0220112. doi: 10.1371/journal.pone.0220112 (PMC6685619; doi:10.1371/journal.pone.0220112)
Supplement: S2 Text — (PDF) [file pone.0220112.s002.pdf]

Application form Psychology Research Ethics Committee (PREC)

1. Which researchers from Leiden University are involved in the study?

Prof. dr. A. W. M. Evers
Dr. D. S. Veldhuijzen
Dr. H. van Middendorp
Drs. L. Schakel

2. Please mention any external researchers involved in the study, including their affiliations.

Prof. dr. J. de Houwer; Universiteit Gent, sectie Experimenteel-Klinische en Gezondheidspsychologie
Dr. P. van Dessel; Universiteit Gent, sectie Experimenteel-Klinische en Gezondheidspsychologie

3. Who is the principal investigator for the study (name and e-mail address)?
   Please make sure that this person is mentioned as a contact person in the information letter belonging to the informed consent and possible debriefing.

Prof. dr. A. W. M. Evers
a.evers@fsw.leidenuniv.nl

4. At which location will the study be performed?
   e.g., at Leiden University, at another institution, or online

Universiteit Leiden, Faculteit der Sociale Wetenschappen

5. Does this application entail an umbrella protocol summarizing different projects, of which some may become more concrete in the future?
   In case of an umbrella protocol, make sure that the protocol is clearly defined around one main theme, including clearly stating the boundaries within which all projects will be conducted regarding ethical issues.

Nee

6. Does this application entail a small change with regard to a previously approved application? Please mention the change here.
   E.g., research with the exact same procedure but a different research group, or other instrument(s)? Or a small change in a task?

Nee

If yes, please describe change.

7. Title of the study:

De invloed van verbale suggesties op de effecten van een ontspanningsoefening ten aanzien van acute stress.

8. Participants: inclusion and exclusion criteria
   e.g., “Only female participants; between 18-30 years of age…”. In case of a study in ‘vulnerable’ groups (e.g., children < 16 years or persons unable to give consent), please take note of the guidelines on our website.

Zowel mannen als vrouwen die tussen de 18 en 35 jaar oud zijn en vloeiend Nederlands spreken mogen deelnemen aan het onderzoek. Proefpersonen met ernstige somatische en/of psychische aandoeningen die interfereren met het studieprotocol, regelmatig drugsgebruik, regelmatig drankgebruik (meer dan 3 eenheden alcohol per dag), aanwezigheid van huidige of recente (< 3 maanden) life events worden uitgesloten van deelname.

9. Number of participants:

In totaal zullen 120 deelnemers worden geworven, gelijkmatig verdeeld over vier condities.

10. Background, reasons for study
    e.g., “Previous research has shown…”

Uit voorgaand onderzoek blijkt dat een korte stressmanagement training positieve effecten kan hebben op zowel de psychologische als fysiologische respons ten aanzien van stress (de Brouwer et al., 2011; Cruess et al., 2015; Gaab et al., 2003; Rosenkranz et al., 2013). De onderliggende mechanismen die een rol spelen bij de effecten van een korte stressmanagement training op het omgaan met stress zijn nog niet duidelijk. Ook is nog niet nader onderzocht in hoeverre een instructie/verbale suggestie over de effecten van een korte stressmanagement training dezelfde effecten teweeg kunnen brengen als het daadwerkelijk uitvoeren van de training. Uit voorgaand onderzoek naar de onderliggende mechanismen van evaluatief conditioneren blijkt dat het geven van een instructie over het benaderen en vermijden van stimuli (bijvoorbeeld bepaalde sociale groepen) dezelfde effecten teweeg kan brengen als het daadwerkelijk uitvoeren van een training gericht op het benaderen en vermijden van stimuli (Van Dessel, De Houwer, Gast, & Tucker Smith, 2015). Echter is nog niet onderzocht in hoeverre deze resultaten op het gebied van instructies/verbale suggesties te generaliseren zijn naar andere trainingen, zoals bijvoorbeeld een korte stressmanagement training. Daarom is het doel van de de huidige studie om te onderzoeken in hoeverre verbale suggesties invloed kunnen hebben op de effecten van een ontspanningsoefening ten aanzien van zelfgerapporteerde stress na blootstelling aan een acute stress taak in gezonde studenten.

11. Research question
    e.g., “The goal of the current study is…”

Het doel van de huidige studie is om te onderzoeken in hoeverre verbale suggesties invloed kunnen hebben op de effecten van een ontspanningsoefening ten aanzien van zelfgerapporteerde stress na blootstelling aan een acute stress taak.

12. Study design
    e.g., “2 x 2 within/ between subjects design”. Explain for example how many conditions your study contains, which manipulations you use, etc.

Een gerandomiseerde experimentele studie zal worden uitgevoerd met een between-subjects design. Proefpersonen worden gerandomiseerd naar één van de vier condities. Conditie 1 is een controle conditie waarin men neutrale filler taken voltooid (puzzels), in conditie 2 krijgt men een stressmanagement training gedurende 25 minuten, bestaande uit twee ontspanningsoefeningen met een korte pauze ertussen, in conditie 3 wordt een instructie/verbale suggestie over de werkzaamheid van ontspanningsoefeningen aan deelnemers gegeven, maar zal men geen ontspanningsoefeningen krijgen en in conditie 4 wordt een instructie/verbale suggestie gegeven en krijgt men ook de ontspanningsoefening.

13. Procedure, instruments & tasks
    e.g., “To assess mood, participants will first complete the Positive and Negative Affect Schedule (PANAS), followed by a manipulation/intervention and a Flanker’s task…”. Please mention all the steps and tasks in your study, including the required time investment by the participant, to enable the PERC to assess participant investment in terms of time and impact.

De procedure van de studie is hieronder weergegeven en wordt daarna nader toegelicht:

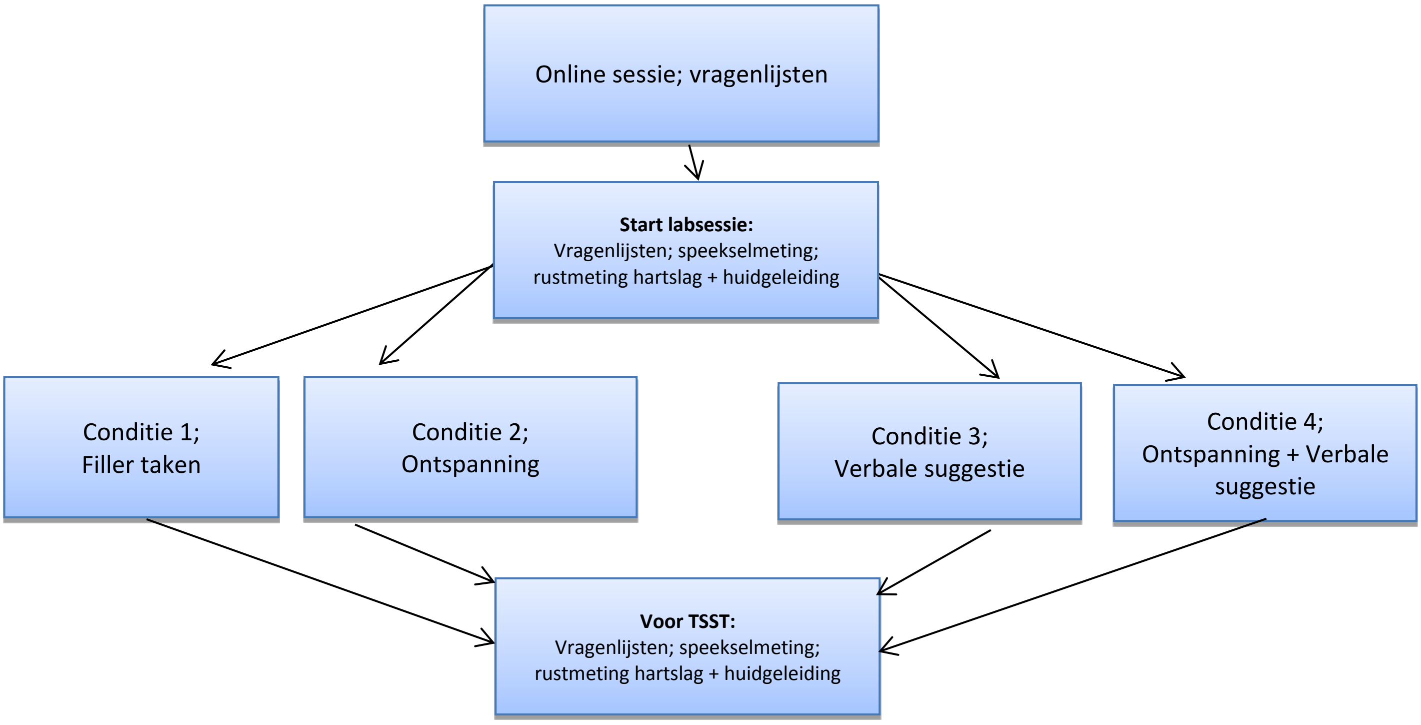

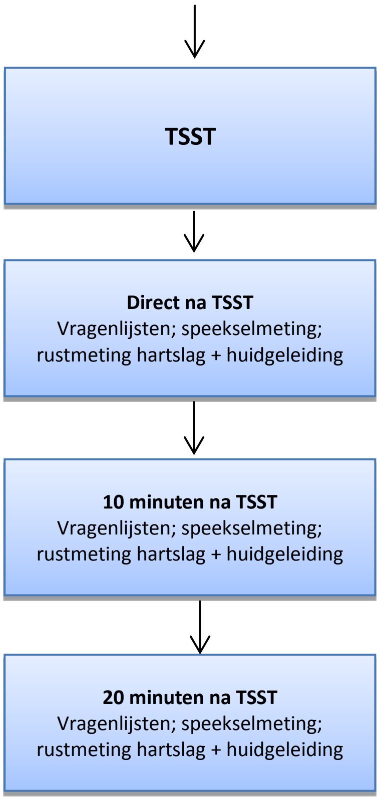

<u>Online sessie; vragenlijsten</u>
Een aantal persoonlijkheidsfactoren zal worden meegenomen, zoals optimisme en pessimisme (gemeten met de Penn State Worry Questionnaire; PSWQ) (Meyer, Miller, Metzger, & Borkovec, 1990), neuroticisme en extraversie (gemeten met de Eysenck Personality Questionnaire – Revised; EPQ-R) (Sanderman, 1995), evenals de neiging tot gedragsinhibitie en gedragsactivatie (gemeten met de Behavioral Inhibition/Behavioral Activation System Scale; BIS/BAS Scale) (Franken, Muris, & Rassin, 2005). Daarnaast worden een aantal vragen afgenomen rondom demografische gegevens om onder andere te kunnen kijken of men voldoet aan de in/exclusiecriteria.

<u>Start labsessie;</u>

a. <u>vragenlijsten</u>

De Shortened State-Trait Anxiety Inventory State version (STAI-S-s; Spielberger et al., 1983) wordt meegenomen om state anxiety te meten; dit is de primaire uitkomst van deze studie. Daarnaast wordt de Numeric Rating Scale (NRS) welzijn (de Brouwer et al., 2011) afgenomen om psychologisch welzijn te meten. Ook wordt de Positive and Negative Affect Schedule (PANAS; Watson et al., 1988) afgenomen om positief en negatief affect te meten. Daarnaast wordt een Visual Analogue Scale (VAS) afgenomen om spanning te meten (de Brouwer et al., 2011). Tot slot wordt de Perceived Stress Scale (PSS: Cohen et al., 1983; Cohen et al., 1988) afgenomen voor het meten van de perceptie van stress.

b. <u>fysiologische maten</u>

Een eerste speekselmonster zal worden afgenomen om niveaus van cortisol en alfa amylase te bepalen.
Daarnaast wordt tijdens de gehele sessie hartslag en huidgeleiding gemeten. Als baseline meting wordt een rustmeting gedaan van de hartslag en huidgeleiding.

Condities
Na het invullen van de vragenlijsten worden proefpersonen gerandomiseerd naar 1 van de 4 condities zoals hierboven beschreven.

In conditie 1 krijgt men filler taken te doen die niets te maken hebben met ontspanning. Proefpersonen zullen woordzoeker puzzels maken gedurende 25 minuten. In conditie 2 krijgt men een korte stressmanagement training. Deze training zal bestaan uit twee ontspanningsoefeningen met een korte pauze tussendoor.
De verbale suggestie die aan personen in conditie 3 en 4 wordt gegeven zal benadrukken dat het doen van ontspanningsoefeningen effectief is gebleken in het leren omgaan met stress. In conditie 4 wordt tevens de korte stress manamgement training aangeboden.

Voor TSST
Voor de TSST zullen vragenlijsten worden ingevuld, namelijk de STAI-S-s, PANAS, NRS en VAS. Ook zal een rustmeting worden gedaan van de hartslag en huidgeleiding en zal een tweede speekselmonster worden afgenomen voor het meten van cortisol en alfa amylase.

TSST
Alle Proefpersonen zullen de Trier Social Stress Test (TSST; Kirschbaum et al., 1993) ondergaan. Deze test bestaat uit een voorbereide presentatie over hun droomberoep gedurende 6 minuten, waarvoor men eerst 5 minuten voorbereidingstijd krijgt, en een hoofdrekentaak van 4 minuten. Deelnemers zitten voor een tweekoppige jury die negatieve feedback geeft gedurende de taken. De TSST heeft aangetoond op betrouwbare wijze psychofysiologische reacties teweeg te brengen, inclusief verhoogde cortisolniveaus en sympathische activatie (Denson et al., 2009; Dickerson & Kemeny, 2004).

Direct na TSST
Na de TSST zullen vragenlijsten worden ingevuld, namelijk de STAI-S-s, PANAS, NRS en VAS. Ook zal een rustmeting worden gedaan van de hartslag en huidgeleiding en zal een derde speekselmonster worden afgenomen voor het meten van cortisol en alfa amylase.

10 minuten na TSST
10 minuten na de TSST zullen weer een aantal vragenlijsten worden ingevuld, namelijk de STAI-S-s, PANAS, NRS en VAS. Ook zal weer een rustmeting worden gedaan van de hartslag en huidgeleiding en zal een vierde speekselmonster worden afgenomen voor het meten van cortisol en alfa amylase.

20 minuten na TSST
20 minuten daarna zullen de vragenlijsten opnieuw worden ingevuld (STAI-S-s, PANAS, NRS en VAS). Daarnaast zal een vragenlijst worden ingevuld aangaande psychologische reactantie, namelijk de Hong Psychological Reactance Scale (HPRS; Hong & Faedda, 1996) en wordt een vraag ingevuld rondom demand compliance. Ook zal weer een rustmeting worden gedaan van de hartslag en huidgeleiding en zal een vijfde en laatste speekselmonster worden afgenomen voor het meten van cortisol en alfa amylase.

Debriefing

Na afloop van het experiment zullen deelnemers een uitgebreide debriefing ontvangen over het daadwerkelijke doel van het experiment.

Literatuur

Carver, C. S., & White, T. L. (1994). Behavioral inhibition, behavioral activation, and affective responses to impending reward and punishment: The BIS/BAS scales. *Journal of Personality and Social Psychology*, 67, 319-333.

Cohen, S., Kamarck, T., & Mermelstein, R. (1983). A global measure of perceived stress. *Journal of Health and Social Behavior*, 24, 386-396.

Cohen, S. & Williamson, G. (1988). *Perceived stress in a probability sample of the United States*. In S. Spacapan, & S. Oskamp (Eds.), The Social Psychology of Health. Newbury Park, CA: Sage.

Cruess, D.G., Finitsis, D.J., Smith, A.L., Goshe, B.M., Burnham, K., Burbridge, C., & O'Leary, K. (2015). Brief stress management reduces acute distress and buffers physiological response to a social stress test. *International Journal of Stress Management, 22*, 270-286.

de Brouwer, S.J.M., Kraaimaat, F.W., Sweep, F.C.G.J., Donders, R.T., Eijsbouts, A., van Koulil, S., van Riel, P.L.C.M., & Evers, A.W.M. (2011). Psychophysiological responses to stress after stress management training in patients with rheumatoid arthritis. *PLoS One, 6*, doi: 10.1371/journal.pone.0027432.

Dickerson, S.S. & Kemeny, M.E. (2004). Acute stressors and cortisol responses: A theoretical integration and synthesis of laboratory research. *Psychological Bulletin*, *130*, 355-391.

Eynsenck, S.B.G., Eynsenck, H., & Barrett, P. (1985). A revised version of the Psychoticism scale. *Personality and Individual Differences, 6*, 21-29.

Gaab, J., Blättler, N., Menzi, T., Pabst, B., Stoyer, S., and Ehlert, U. (2003). Randomized controlled evaluation of the effects of cognitive-behavioral stress management on cortisol responses to acute stress in healthy subjects. *Psychoneuroendocrinology, 28*, 767-779.

Hong, S.M., & Faedda, S. (1996). Refinement of the Hong psychological reactance scale. *Educational and Psychological Measurement, 56*, 173-182.

Kirschbaum, C., Pirke, K. M., & Hellhammer, D. H. (1993). The 'Trier Social Stress Test'–a tool for investigating psychobiological stress responses in a laboratory setting. *Neuropsychobiology*, *28*, 76-81.

Kirschbaum, C., Pirke, K.M., & Hellhammer, D.H. (1995). Preliminary evidence for reduced cortisol responsivity to psychological stress in women using oral contraceptive medication. *Psychoneuroendocrinology, 20*, 509-514.

Rosenkranz, M.A., Davidson, R.J., MacCoon, D.G., Sheridan, J.F., Kalin, N.H., & Lutz, A. (2013). A comparison of mindfulness-based stress reduction and an active control in modulation of neurogenic inflammation. *Brain, Behavior and Immunity, 27*, 174-184.

Scheier, M., Carver, C.S., & Bridges, M.W. (1994). Distinguishing optimism from neuroticism (and trait anxiety, self-mastery, and self-esteem): a re-evaluation of the Life Orientation Test. *Journal of Personality and Social Psychology, 67*, 1063-1078.

Spielberger, C., Gorsuch, R.L., Lushene, R., Vagg, P.R., & Jacobs, G.A. (1983). *Manual for the State-Trait Anxiety Inventory.* Palo Alto, CA: Consulting Psychologists Press.

Van Dessel, P., De Houwer, J., Gast, A., & Tucker Smith, C. (2015). Instruction-Based Approach-Avoidance Effects: Changing Stimulus Evaluation via the Mere Instruction to Approach or Avoid Stimuli. *Experimental Psychology, 62,* 161-169.

Watson, D., Clark, L.A., & Tellegen, A. (1988). Development and validation of brief measures of positive and negative affect:the PANAS Scales. *Journal of Personality and Social Psychology, 54*, 1063-1070.

14. Are participants compensated for their participation?
    Please take note of the guidelines on our website.

Ja; volgens het standaard uurtarief

In case of deviation from standard compensation, please explain here.

15. Does the study include psychophysiological assessments?
    Please take note of the guidelines on our website.

Ja: speekselmonsters worden afgenomen om niveaus van cortisol en alfa amylase te bepalen op verschillende tijdsmomenten tijdens de sessie. Daarnaast worden hartslag en huidgeleiding continu op discrete wijze gemeten door middel van Biopac apparatuur.

16. Does the study entail deception?
    e.g., by providing purposefully incorrect or incomplete information about the aim of the study or by incorrect feedback about the experimental manipulations. Please take note of the guidelines on our website.

Ja; aan proefpersonen wordt verteld dat de studie gaat over de relatie tussen aandachtsprocessen en rekenvaardigheden. Proefpersonen worden er niet van op de hoogte gebracht dat er vier verschillende condities zijn en dat in twee van deze condities verbale suggesties worden gegeven aan proefpersonen. Aan het eind van het onderzoek ontvangt iedere deelnemer een uitgebreide debriefing over het daadwerkelijke doel van het onderzoek.

17. Are 'unobtrusive methods' used in the study?
    I.e., data being collected, e.g., observing or videotaping the behavior of the participant, without the participant being informed? Please take note of the guidelines on our website.

Nee

18. Are participants fully debriefed about the deception and/or use of unobtrusive methods after the study?
    Please take note of the guidelines on our website.

Ja

If no, please explain here.

19. Will the data be collected anonymously or processed in a coded way? Please take note of the guidelines on our website.

Ja; dataverzameling en verwerking zal plaatsvinden op een gecodeerde manier. Anonieme proefpersoon identificatie codes zullen worden gebruikt om de data te linken aan de proefpersoon. Het bestand wat toegang geeft tot de link tussen de proefpersoon en de persoonlijke data (bijvoorbeeld de naam van de proefpersoon), zal worden bijgehouden door het onderzoeksteam. Het onderzoeksteam heeft als enige toegang tot dit bestand.

If not collected anonymously and saved in a coded way, please explain here.

20. Below, paste your Informed Consent form.
    For examples, see the guidelines on our website.

Informatie voor Deelnemers
Onderzoek naar aandachtsprocessen en rekenvaardigheden

Geachte heer/mevrouw,

Op dit moment is de sectie Gezondheids-, Medische en Neuropsychologie van de Universiteit Leiden op zoek naar gezonde vrijwilligers die willen deelnemen aan een onderzoek waarin de relatie tussen aandachtsprocessen en rekenvaardigheden wordt getoetst. Door middel van deze informatiebrief willen we u meer uitleg geven over dit onderzoek en u uitnodigen voor deelname. Lees deze informatiebrief rustig door voordat u besluit of u wel of niet deel wilt nemen.

Doel van het onderzoek
Het doel van het onderzoek is om de relatie tussen aandachtsprocessen en rekenvaardigheden te onderzoeken. We vragen u om een aantal vragenlijsten in te vullen en verschillende taken voltooien. Daarnaast zullen we fysiologische meetapparatuur gebruiken om uw hartslag en huidgeleiding te meten en zullen we u ook vragen om speeksel af te geven.

Wat wordt er van u gevraagd?
Indien u instemt met deelname aan het onderzoek, zal u eerst online een aantal vragenlijsten invullen. Dit zal ongeveer een kwartier duren. Daarna zal u eenmalig worden uitgenodigd op de Faculteit der Sociale Wetenschappen van de Universiteit Leiden om nog een aantal vragenlijsten in te vullen en een aantal taken te doen, zo zal u onder andere een rekentaak onder tijdsdruk uit voeren, welke kan zorgen voor een kortdurende toename van psychofysiologische reacties. Daarnaast zal u speeksel af geven en zal uw hartslag en huidgeleiding worden gemeten met een aantal sensoren op uw huid. Het onderzoek zal een totale tijdsinvestering van ongeveer 2 uur van u vragen.

Belangrijk! Om de metingen zo zuiver mogelijk uit te voeren, vragen we u om 2 uur voor de beide sessies geen koffie, thee, cola of energydrank te nuttigen en geen zware (warme) maaltijd te eten. Daarnaast mag u op de avond voor de afspraak niet sporten, geen alcohol drinken of drugs gebruiken.

Wie mag deelnemen?
Iedereen tussen de 18 en 35 jaar oud die vloeiend Nederlands spreekt en geen last heeft

van chronische lichamelijke aandoeningen en/of psychische klachten kan aan dit onderzoek deelnemen. Overmatige drugs- en alcoholgebruikers kunnen niet meedoen aan dit onderzoek.

Mogelijke voor- en nadelen van de deelname
U heeft zelf geen voordeel van deelname aan deze onderzoeken. Er zijn ook geen risico's bekend voor deelname aan deze onderzoeken. Alle gebruikte testen en procedures in deze onderzoeken zijn onschadelijk. Het enige mogelijke nadeel van deelname is de tijdsinvestering die van u gevraagd wordt.

Ethische toetsing
Dit onderzoek is goedgekeurd door de Commissie Ethiek Psychologie van het Instituut Psychologie, Universiteit Leiden.

Vrijwilligheid van de deelname
Deelname aan dit onderzoek is uiteraard geheel vrijwillig. U kunt dus besluiten om niet mee te doen of op elk moment met de deelname aan dit onderzoek stoppen. Dit zal geen negatieve gevolgen hebben voor u en u hoeft daarvoor geen redenen aan te geven. U wordt naar ratio van tijdsinvestering uitbetaald.

Gegevens & Vertrouwelijkheid
De persoonlijke gegevens die tijdens het onderzoek worden verzameld worden geheel vertrouwelijk behandeld en anoniem verwerkt. Uw onderzoeksgegevens worden door ons gedurende 15 jaar bewaard. De personen die inzage hebben in de door u aan ons verstrekte gegevens zijn de onderzoeksmedewerkers.

Vergoeding
Bij deelname aan dit onderzoek ontvangt u € 15,- contant of 4 credits als tegemoetkoming voor de tijdsbesteding.

Wilt u deelnemen?
Indien u interesse heeft om deel te nemen aan deze onderzoeken, kunt zich via SONA (http://ul.sona-systems.com) aanmelden of e-mailen naar rekenonderzoek@fsw.leidenuniv.nl.

Klachten
Indien u klachten heeft over deze onderzoeken, kunt u deze bespreken met de uitvoerende onderzoeker (in persoon of via het email adres hieronder vermeld) of u kunt contact opnemen met de projectleider, mevr. Prof. Dr. A. W. M. Evers (via secretariaat, telefoonnummer 071-527 3627).

Vragen
Heeft u vragen over deze onderzoeken, twijfelt u over deelname of wilt u meer informatie? Dan kunt u contact opnemen door een e-mail te sturen naar rekenonderzoek@fsw.leidenuniv.nl.

Met vriendelijke groeten,

Mw. L. Schakel, MSc
Mw. Prof. dr. A.W.M. Evers

Toestemmingsverklaring
Aandachtsprocessen en rekenvaardigheden

Voor de deelnemer:
Ik ben naar tevredenheid over dit onderzoek geïnformeerd. Ik heb schriftelijke informatie gekregen en ben in de gelegenheid gesteld om vragen over het onderzoek te stellen. Ik begrijp dat deelname aan het onderzoek geheel vrijwillig is en dat ik op ieder moment kan stoppen met het onderzoek zonder dat ik daarvoor een reden hoef op te geven. Ik weet dat mijn gegevens gecodeerd worden verwerkt. Ik geef toestemming om mijn gegevens te gebruiken, voor de doelen die in de informatiebrief staan.

☐ Ik ben bereid deel te nemen aan dit onderzoek.

☐ Ik geef mijn toestemming om voor vervolgonderzoek benaderd te worden, na afloop van dit onderzoek.

Hierbij stem ik toe met deelname aan dit onderzoek:

Naam: ……………………………………………

Geboortedatum: ………………………………

Datum: ……/……/………… Handtekening: ……………………………

Voor de onderzoeker:
Ondergetekende verklaart dat de hierboven genoemde persoon volledig over bovengenoemd
onderzoek is geïnformeerd. Zij/hij verklaart tevens dat een voortijdige beëindiging van deelname van de bovengenoemde persoon op geen enkele wijze nadelige gevolgen zal hebben voor deze persoon.

Naam: ……………………………………………

Datum: ……/……/………… Handtekening: ……………………………

21. Below, paste your Debriefing.
    For examples, see the guidelines on our website.

Algemene debriefing
Graag zou ik je nog wat meer over het onderzoek vertellen. Wat we je van tevoren niet konden vertellen, omdat het mogelijk jouw reactie op de rekentaak zou kunnen beïnvloeden,

is dat ons onderzoek specifiek gaat over het effect van een verbale suggestie die gerelateerd is aan een stressmanagement training op verschillende psychologische en fysiologische uitkomstmaten na stress. De presentatie en de rekentaak waren dan ook geen taken naar je presentatie- en rekenvaardigheden, maar waren bedoeld om je stressniveau te verhogen. Ik kan me voorstellen dat je die taak hebt ervaren als onprettig, maar het is belangrijk om te weten dat de reactie van de commissie op jouw presentatie en jouw rekenvaardigheden niet realistisch was. De taak is namelijk speciaal ontwikkeld om vervelende gevoelens op te wekken. De commissieleden reageren dus op iedereen op dezelfde manier en dat heeft niets te maken met jouw competenties. In werkelijkheid zijn de commissieleden vriendelijke personen.

Dit onderzoek bestond uit 4 condities, namelijk een controlegroep waarin woordzoeker puzzels werden aangeboden, een groep waarin een ontspanningsoefening werd aangeboden, een groep waarin de instructie werd gegeven dat een ontspanningsoefening effectief is en dat de ontspanningsoefening later gegeven zouden worden, maar deze is uiteindelijk niet meer gegeven en een laatste conditie waarin men de instructie kreeg dat een ontspanningsoefening effectief is en ook de ontspanningsoefening werd geoefend. We wilden met deze verschillende condities namelijk weten wat de effecten van een ontspanningsoefening zijn op het omgaan met stress en de rol van het geven van een suggestie over de effectiviteit van de ontspanningsoefening hierin.

Jij zat in groep *XXX*.

Door middel van de vragenlijsten die je hebt ingevuld, kunnen we naar je subjectieve stressreactie kijken, bijvoorbeeld de invloed van de stressoren op je stemming.
De hartslag- en huidgeleidingsmetingen zijn meer fysieke stressindicatoren waarmee we de effecten van stress kunnen onderzoeken. Ook hebben wij speeksel bij je afgenomen om de effecten op cortisol te onderzoeken. Cortisol is een belangrijk stresshormoon, wat bij langdurige verhoging negatieve gezondheidseffecten zou kunnen hebben. Door het verzamelen van speeksel kunnen we kijken naar de cortisolreactie op de stresstaak. Meedoen aan dit onderzoek heeft voor jou geen langdurige negatieve gevolgen, we weten namelijk dat de stressreactie op deze taak van korte duur is.
Ik wil je trouwens nog vragen of je eerder over dit onderzoek gehoord hebt. Heb je misschien al mensen gesproken die aan dit onderzoek meegedaan hebben?

[zo ja:] Wat heeft deze persoon je verteld?

Ten slotte vraag ik je nadrukkelijk om aan niemand iets over de inhoud van het onderzoek te vertellen, met name niet over de daadwerkelijke onderzoeksvraag en de presentatie- en rekentaken. Dit kan de metingen erg beïnvloeden. Als je nog mensen weet die mee willen doen, dan zijn ze uiteraard van harte welkom, maar vertel aan hen dus niets over de inhoud van het onderzoek wat niet in de advertentie of brief staat, omdat dit de resultaten sterk kan beïnvloeden.

22. Below, paste any other information relevant for ethical evaluation of the application
    e.g., advertorials, declaration of consent by external institution when participants fall under their responsibility.

Click here to enter

Wil jij graag snel 15 euro of 4 credits verdienen?

Vrijwilligers gezocht voor onderzoek naar aandachtsprocessen en rekenvaardigheden

Ben jij tussen de 18 en 35 jaar? Dan zijn wij op zoek naar jou!

De sectie Gezondheids-, Medische en Neuropsychologie van de Universiteit Leiden, zoekt vrijwilligers voor deelname aan een onderzoek naar aandachtsprocessen en rekenvaardigheden.

Voor dit onderzoek zal je eerst online een aantal vragenlijsten invullen. Daarna zal je uitgenodigd worden op de Faculteit der Sociale Wetenschappen om een aantal vragenlijsten in te vullen en verschillende taken te voltooien. Daarnaast zullen we fysiologische meetapparatuur gebruiken om je hartslag en huidgeleiding te meten en zullen we je vragen om speeksel af te geven.
In totaal zal dit onderzoek ongeveer 2 uur duren.

Meer informatie? Mail dan naar rekenonderzoek@fsw.leidenuniv.nl

supplementary information relevant for ethical review.
