# Supplementary material for: Can verbal suggestions strengthen the effects of a relaxation intervention?
Source: PLoS One. 2019 Aug 7;14(8):e0220112. doi: 10.1371/journal.pone.0220112 (PMC6685619; doi:10.1371/journal.pone.0220112)
Supplement: S3 Text — (PDF) [file pone.0220112.s003.pdf]

Application form Psychology Research Ethics Committee (PREC)

1. Which researchers from Leiden University are involved in the study?

Prof. dr. A. W. M. Evers
Dr. D. S. Veldhuijzen
Dr. H. van Middendorp
Drs. L. Schakel

2. Please mention any external researchers involved in the study, including their affiliations.

Prof. dr. J. de Houwer; Ghent University, section Experimental Clinical and Health psychology
Dr. P. van Dessel; Ghent University, section Experimental Clinical and Health psychology

3. Who is the principal investigator for the study (name and e-mail address)?
   Please make sure that this person is mentioned as a contact person in the information letter belonging to the informed consent and possible debriefing.

Prof. dr. A. W. M. Evers
a.evers@fsw.leidenuniv.nl

4. At which location will the study be performed?
   e.g., at Leiden University, at another institution, or online

Leiden University, Faculty of Social Sciences

5. Does this application entail an umbrella protocol summarizing different projects, of which some may become more concrete in the future?
   In case of an umbrella protocol, make sure that the protocol is clearly defined around one main theme, including clearly stating the boundaries within which all projects will be conducted regarding ethical issues.

No

6. Does this application entail a small change with regard to a previously approved application? Please mention the change here.
   E.g., research with the exact same procedure but a different research group, or other instrument(s)? Or a small change in a task?

No

If yes, please describe change.

7. Title of the study:

The influence of verbal suggestions on the effects of a relaxation practice regarding acute stress.

8. Participants: inclusion and exclusion criteria
   e.g., “Only female participants; between 18-30 years of age…”. In case of a study in ‘vulnerable’ groups (e.g., children < 16 years or persons unable to give consent), please take note of the guidelines on our website.

Males as well as females between 18 and 35 years of age and who speak Dutch fluently may participate in the study. Participants with severe somatic and/or psychological conditions that interfere with the study protocol, frequent use of drugs, frequent use of alcohol (more than 3 units alcohol a day), presence of current or recent (< 3 months) life events will be excluded from participation.

9. Number of participants:

In total, 120 participants will be recruited, equally allocated over the four conditions.

10. Background, reasons for study
    e.g., “Previous research has shown…”

Previous research showed that a short stress management training can have positive effects on the psychological as well as the physiological response regarding stress (de Brouwer et al., 2011; Cruess et al., 2015; Gaab et al., 2003; Rosenkranz et al., 2013). The underlying mechanisms playing a role in the effects of a short stress management training on coping with stress are not yet elucidated. Additionally, it is not yet investigated whether an instruction/verbal suggestion concerning the effects of a short stress management training can cause the same effects as actually performing the training. From previous research on the underlying mechanisms of evaluative conditioning it is known that providing an instruction on approaching and avoiding stimuli (e.g, certain social groups) can lead to the same effects as actually performing a training directed at approaching and avoiding stimuli (Van Dessel, De Houwer, Gast, & Tucker Smith, 2015). However, it is not yet investigated whether the results in the area of instructions/verbal suggestions can be generalized to other trainings, such as a short stress management training. Therefore, the aim of the present study is to investigate whether verbal suggestions can influence the effects of a relaxation practice regarding self-reported stress after exposure to an acute stress challenge in healthy students.

11. Research question
    e.g., “The goal of the current study is…”

The goal of the current study is to investigate whether verbal suggestions can influence the effects of a relaxation practice regarding self-reported stress after exposure to an acute stress challenge.

12. Study design
    e.g., “2 x 2 within/ between subjects design”. Explain for example how many conditions your study contains, which manipulations you use, etc.

A randomized experimental study will be performed with a between-subjects design. Participants will be randomized to one of four conditions. Condition 1 is a control condition in which neutral filler task are completed (puzzles), in condition 2, participants receive a stress management training during 25 minutes, consisting of two relaxation practices with a short break in between, in condition 3, an instruction/verbal suggestion concerning the efficacy of relaxation practices will be given to participants, but they will not receive the relaxation practices and in condition 4, an instruction/verbal suggestion will be given and also the relaxation practice.

13. Procedure, instruments & tasks
    e.g., “To assess mood, participants will first complete the Positive and Negative Affect Schedule (PANAS), followed by a manipulation/intervention and a Flanker’s task…”. Please mention all the steps and tasks in your study, including the required time investment by the participant, to enable the PERC to assess participant investment in terms of time and impact.

The procedure of the study is presented below and will be further explained:

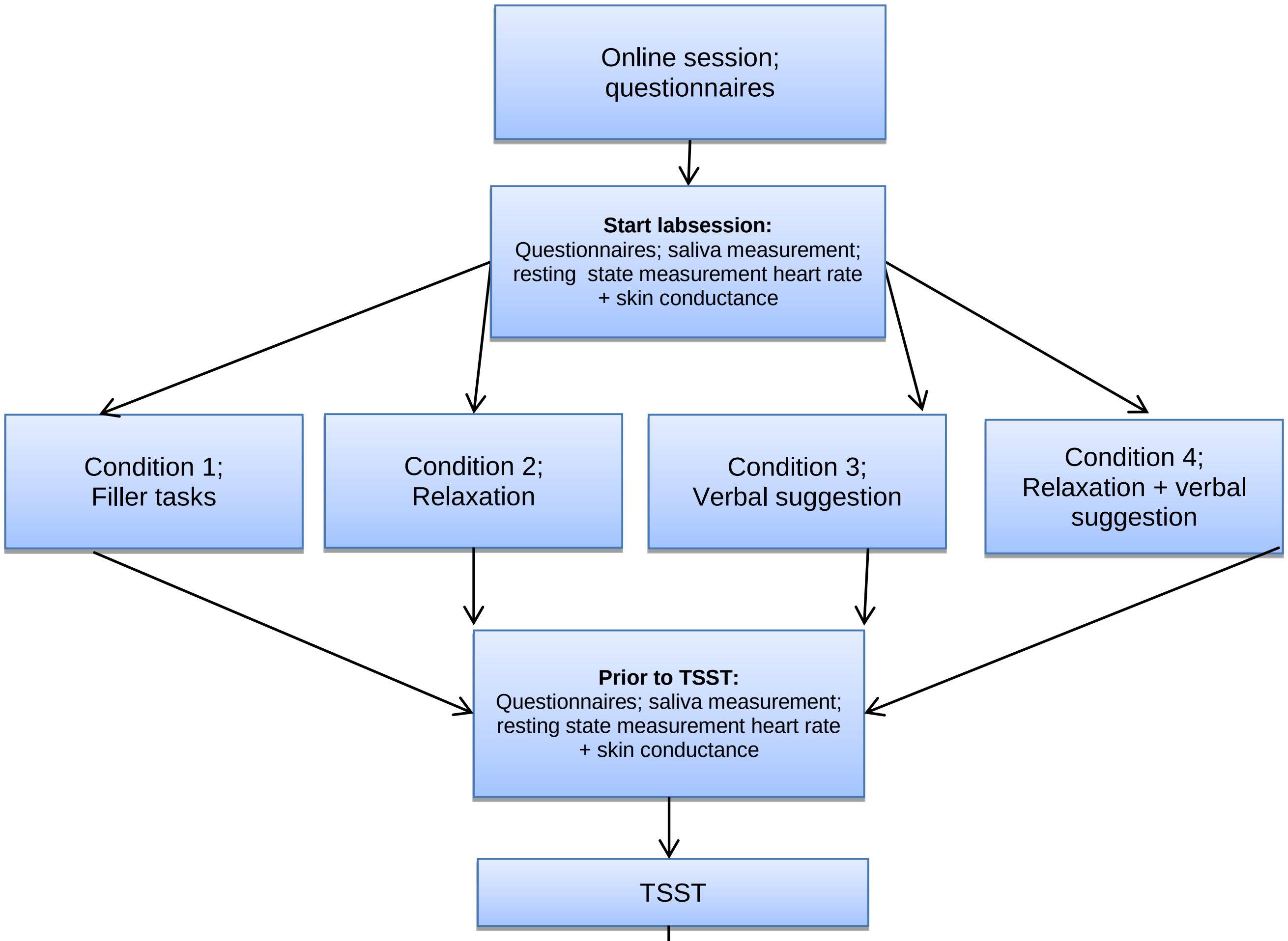

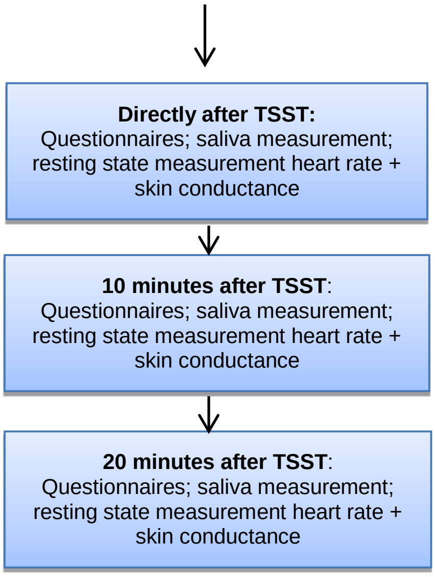

<u>Online session; questionnaires</u>

A couple of personality factors will be taken into account, such as optimism and pessimism (measured with the Penn State Worry Questionnaire; PSWQ) (Meyer, Miller, Metzger, & Borkovec, 1990), neuroticism and extraversion (measured with the Eysenck Personality Questionnaire – Revised; EPQ-R) (Sanderman, 1995), as well as the tendency to behavioral inhibition and behavioral activation (measured with the Behavioral Inhibition/Behavioral Activation System Scale; BIS/BAS Scale) (Franken, Muris, & Rassin, 2005). Besides, a couple of questionnaires concerning demographics will be taken a.o. to see whether people meet the in/exclusion criteria.

<u>Start labsession;</u>

a. <u>questionnaires</u>

The Shortened State-Trait Anxiety Inventory State version (STAI-S-s; Spielberger et al., 1983) will be incorporated to measure state anxiety; this is the primary outcome of this study. In addition, the Numeric Rating Scale (NRS) well-being (de Brouwer et al., 2011) will be taken to measure psychological well-being. Also, the Positive and Negative Affect Schedule (PANAS; Watson et al., 1988) will be taken to measure positive and negative affect. Besides, a Visual Analogue Scale (VAS) will be taken to measure tension (de Brouwer et al., 2011). Finally, the Perceived Stress Scale (PSS: Cohen et al., 1983; Cohen et al., 1988) will be taken to measure the perception of stress.

b. <u>physiological measures</u>

A first saliva sample will be taken to determine levels of cortisol and alpha amlyase. Besides, heart rate and skin conduction will be measured during the whole session. As a baseline measure, a resting state measurement of heart rate and skin conductance will be performed.

<u>Conditions</u>

After completing the questionnaires, participants will be randomized to 1 of 4 conditions as described above.

In condition 1, participants receive filler tasks that are unrelated to relaxation. Participants will complete wordfinding puzzles during 25 minutes. In condition 2, participants receive a short stress management training. This training will exist of 2 relaxation practices with a short break in between.
The verbal suggestion that is given to participants in condition 3 and 4 will emphasize that doing relaxation practices is effective in learning to cope with stress. In condition 4, the short stress management training will also be provided.

Prior to TSST

Prior to the TSST questionnaires will be completed, including the STAI-S-s, PANAS, NRS and VAS. In addition, a resting state measurement of heart rate and skin conductance will be completed, as well as a second saliva sample to measure cortisol and alpha amylase.

TSST

All participants will be exposed to the Trier Social Stress Test (TSST; Kirschbaum et al., 1993). This test consists of a prepared presentation about their dream job during 6 minutes, for which they first receive 5 minutes preparation time, and an arithmetic task by head of 4 minutes. Participants sit in front of a twoheaded jury that provide negative feedback during the tasks. The TSST has demonstrated to reliably activate psychophysiological reactions, including heightened cortisol levels and symphathetic activation (Denson et al., 2009; Dickerson & Kemeny, 2004).

Directly after TSST

After the TSST, questionnaires will be completed: the STAI-S-s, PANAS, NRS and VAS. In addition, a resting state measurement of heart rate and skin conductance will be completed, as well as a third saliva sample to measure cortisol and alpha amylase.

10 minutes after TSST

10 minutes after TSST, again a couple of questionnaires will be completed: the STAI-S-s, PANAS, NRS and VAS. Also, a resting state measurement of heart rate and skin conductance will be completed again, and a fourth saliva sample will be taken to measure cortisol and alpha amylase.

20 minutes after TSST

20 minutes afterwards, the questionnaires will be completed again (STAI-S-s, PANAS, NRS and VAS). In addition, a questionnaire will be filled out regarding psychological reactance: the Hong Psychological Reactance Scale (HPRS; Hong & Faedda, 1996) and a question will be filled out regarding demand compliance. In addition, a resting state measurement of heart rate and skin conductance will be completed again, and a fifth and final saliva sample will be taken to measure cortisol and alpha amylase.

Debriefing

At the end of the experiment, participants will receive an extensive debriefing concerning the actual aim of the experiment.

Literature

Carver, C. S., & White, T. L. (1994). Behavioral inhibition, behavioral activation, and affective responses to impending reward and punishment: The BIS/BAS scales. *Journal of Personality and Social Psychology*, 67, 319-333.

Cohen, S., Kamarck, T., & Mermelstein, R. (1983). A global measure of perceived stress. *Journal of Health and Social Behavior*, 24, 386-396.

Cohen, S. & Williamson, G. (1988). *Perceived stress in a probability sample of the United*

States. In S. Spacapan, & S. Oskamp (Eds.), The Social Psychology of Health. Newbury Park, CA: Sage.
Cruess, D.G., Finitsis, D.J., Smith, A.L., Goshe, B.M., Burnham, K., Burbridge, C., & O'Leary, K. (2015). Brief stress management reduces acute distress and buffers physiological response to a social stress test. *International Journal of Stress Management, 22*, 270-286.
de Brouwer, S.J.M., Kraaimaat, F.W., Sweep, F.C.G.J., Donders, R.T., Eijsbouts, A., van Koulil, S., van Riel, P.L.C.M., & Evers, A.W.M. (2011). Psychophysiological responses to stress after stress management training in patients with rheumatoid arthritis. *PLoS One, 6*, doi: 10.1371/journal.pone.0027432.
Dickerson, S.S. & Kemeny, M.E. (2004). Acute stressors and cortisol responses: A theoretical integration and synthesis of laboratory research. *Psychological Bulletin*, *130*, 355-391.
Eynsenck, S.B.G., Eynsenck, H., & Barrett, P. (1985). A revised version of the Psychoticism scale. *Personality and Individual Differences, 6*, 21-29.
Gaab, J., Blättler, N., Menzi, T., Pabst, B., Stoyer, S., and Ehlert, U. (2003). Randomized controlled evaluation of the effects of cognitive-behavioral stress management on cortisol responses to acute stress in healthy subjects. *Psychoneuroendocrinology, 28*, 767-779.
Hong, S.M., & Faedda, S. (1996). Refinement of the Hong psychological reactance scale. *Educational and Psychological Measurement, 56*, 173-182.
Kirschbaum, C., Pirke, K. M., & Hellhammer, D. H. (1993). The 'Trier Social Stress Test'–a tool for investigating psychobiological stress responses in a laboratory setting. *Neuropsychobiology*, *28*, 76-81.
Kirschbaum, C., Pirke, K.M., & Hellhammer, D.H. (1995). Preliminary evidence for reduced cortisol responsivity to psychological stress in women using oral contraceptive medication. *Psychoneuroendocrinology, 20*, 509-514.
Rosenkranz, M.A., Davidson, R.J., MacCoon, D.G., Sheridan, J.F., Kalin, N.H., & Lutz, A. (2013). A comparison of mindfulness-based stress reduction and an active control in modulation of neurogenic inflammation. *Brain, Behavior and Immunity, 27*, 174-184.
Scheier, M., Carver, C.S., & Bridges, M.W. (1994). Distinguishing optimism from neuroticism (and trait anxiety, self-mastery, and self-esteem): a re-evaluation of the Life Orientation Test. *Journal of Personality and Social Psychology, 67*, 1063-1078.
Spielberger, C., Gorsuch, R.L., Lushene, R., Vagg, P.R., & Jacobs, G.A. (1983). *Manual for the State-Trait Anxiety Inventory.* Palo Alto, CA: Consulting Psychologists Press.
Van Dessel, P., De Houwer, J., Gast, A., & Tucker Smith, C. (2015). Instruction-Based Approach-Avoidance Effects: Changing Stimulus Evaluation via the Mere Instruction to Approach or Avoid Stimuli. *Experimental Psychology, 62,* 161-169.
Watson, D., Clark, L.A., & Tellegen, A. (1988). Development and validation of brief measures of positive and negative affect:the PANAS Scales. *Journal of Personality and Social Psychology, 54*, 1063-1070.

14. Are participants compensated for their participation?
    Please take note of the guidelines on our website.

Yes; according to the standard hourly rate

In case of deviation from standard compensation, please explain here.

15. Does the study include psychophysiological assessments?
    Please take note of the guidelines on our website.

Yes: saliva samples will be taken to determine levels of cortisol and alpha amylase on various time points during the session. In addition, heart rate and skin conductance is measured continuously in a discrete way by means of Biopac equipment.

16. Does the study entail deception?
    e.g., by providing purposefully incorrect or incomplete information about the aim of the study or by incorrect feedback about the experimental manipulations. Please take note of the guidelines on our website.

Yes; participants are told that the study is about the relation between attentional processes and arithmetic skills. Participants are not informed that there are four different conditions and that in two of those conditions verbal suggestions will be given to participants. At the end of the study, every participant receives an extensive debriefing concerning the actual study aim.

17. Are ‘unobtrusive methods’ used in the study?
    I.e., data being collected, e.g., observing or videotaping the behavior of the participant, without the participant being informed? Please take note of the guidelines on our website.

No

18. Are participants fully debriefed about the deception and/or use of unobtrusive methods after the study?
    Please take note of the guidelines on our website.

Yes

If no, please explain here.

19. Will the data be collected anonymously or processed in a coded way? Please take note of the guidelines on our website.

Yes; data collection and processing will take place in a coded manner. Anonymous participant identification codes will be used to link the data to the participant. The file that

provides access to the link between the participant and personal data (e.g., the name of the participant), will be tracked by the research team. Only the research team has access to this file.

If not collected anonymously and saved in a coded way, please explain here.

20. Below, paste your Informed Consent form.
    For examples, see the guidelines on our website.

Information for Participants
Research on attentional processes and arithmetic skills

Dear sir/madam,

At this moment, the section Health, Medical and Neuropsychology of Leiden University is searching for healthy volunteers who will participate in a study in which the relation between attentional processes and arithmetic skills will be tested. By means of this information letter, we will provide more explanation concerning this study and invite you for participation. Please read this information letter carefully before you decide whether you want to participate or not.

Aim of the study
The aim of the study is to investigate the relation between attentional processes and arithmetic skills. We ask you to fill in a couple of questionnaires and to complete several tasks. In addition, we will use physiological measurement devices to measure your heart rate and skin conductance and will ask you to provide saliva.

What will be asked from you?
If you agree with participation to this research, you will first complete a couple of questionnaires online. This will take around 15 minutes. Afterwards, you will be invited once to the Faculty of Social Sciences of Leiden University to again complete a couple of questionnaires and to do a couple of tasks, as you will a.o. perform an arithmetic task with time pressure, which can lead to a short increase of psychophysiological reactions. Besides, you will provide saliva and your heart rate and skin conductance will be measured with a couple of sensors on your skin. The study will ask a total time investment of around two hours from you.

Important! To keep the measurements as pure as possibly, we ask you not to consume coffee, cola or energydrank 2 hours before the session and not to eat a heavy (warm) meal. In addition, you may not perform physical exercise, not consume alcohol or drugs at the night before the appointment.

Who may participate?
Everyone between 18 and 35 years of age who speak Dutch fluenty and does not suffer from chronical somatic conditions and/or psychological conditions can participate in this study. Excessive drugs and alcohol consumers cannot participate to this study.

Possible pros and cons of participation

You do not have benefits from participation to this study by yourself. There are also no risks known from participation to this study. All used tests and procedures in these studies are harmless. The only disadvantage of particpation is the time investment that is asked from you.

Ethical testing
This study is approved by the Ethics Committee of Psychology of the Institute Psychology, Leiden University.

Voluntariness to participate
Participation to this study is of course completely voluntary. You can thus decide not to participate or to stop study participation at every moment. This will have no negative consequences for you and you do not have to provide reasons for that. You will be payed in proportion to the time investment.

Data & Confidentiality
The personal data that will be collected during the study will be treated completely confidential and anonymously processed. Your research data will be saved by us during 15 years. The persons that have access to the data that you provided to us are the research personnel.

Compensation
After participating to this study, you will receive € 15,- in cash or 4 course credits as compensation for your time investment.

Do you want to participate?
If you are interested in participating to this study, you can subscribe via SONA (http://ul.sona-systems.com) or send an e-mail to rekenonderzoek@fsw.leidenuniv.nl.

Complaints
If you have complaints concerning this study, you can discuss them with the executive researcher (in person or via the e-mail address that is stated below) or you can make contact with the project leader, ms. Prof. Dr. A. W. M. Evers (via secretary, phone number 071-527 3627).

Questions
Do you have questions concerning this study, do you hesitate about participation or do you want additional information? Than you can contact by sending an e-mail to rekenonderzoek@fsw.leidenuniv.nl.

Kind regards,

Ms. L. Schakel, MSc
Ms. Prof. dr. A.W.M. Evers

Informed consent
Attentional processes and arithmetic skills

For the participant:
I am satisfactorily informed about this research. I have received written information and I am given the opportunity to ask questions about this study. I understand that participation in this study is completely voluntary and that I can stop with the study at any moment without providing a reason. I know that my data are processed in a coded manner. I give consent to use my data, for the purposes that are written in the information letter.

☐ I give consent to participate to this study.

☐ I give consent to be contacted for future research, after the end of this study.

Hereby I agree with participation to this study:

Name: ……………………………………………

Date of birth: ………………………………

Date: ……/……/………… Signature: ……………………………

For the researcher:
Undersigned declares that the above mentioned person is informed completely about the above stated study. She/he also declares that the premature termination of participation of the above mentioned person will not have any negative consequences for this person.

Name: ……………………………………………

Date: ……/……/………… Signature: ……………………………

21. Below, paste your Debriefing.
    For examples, see the guidelines on our website.

General debriefing
I would like to tell you somewhat more about the study. What we could not tell you beforehand, because it could possibly influence your reactions on the arithmetic task, is that our study specifically is about the effect of a verbal suggestion that is related to a stress management training on several psychological and physiological outcome measures after stress. The presentation and arithmetic task were therefore no tasks concerning your presentation and arithmetic skills, but were meant to increase your stress level. I can imagine that you have experienced this task as being unpleasant, but it is important to know that the reaction of the committee on your presentation and your arithmetic skills was not realistic. The tasks is specifically developed to provoke unpleasant feelings. The committee members thus react in a same manner to everyone and that has nothing to do with your competences. In real, the committee members are friendly persons.

This study consisted of 4 conditions, namely a control group in which word finding puzzles were provided, a group in which a relaxation practice was provided, a group in which the instruction was provded that a relaxation practice is effective and that the relaxation practice would be provided later, but this was not given anymore at the end and a final condition in which people received the instruction that a relaxation practice was effective and the relaxation practice was also practiced. With those different conditions, we wanted to know what the effects of a relaxation practice are on coping with stress and the role of providing a verbal suggestion on the effectiveness of a relaxation practice hereby.

You were in condition *XXX*.

By means of questionnaires that you have filled out, we can look at your subjective stress reaction, for example the influence of stressors on your mood. The heart rate and skin conductance measurements are rather physical indicators of stress with which we can investigate the effects of stress. We also took saliva from you to investigate the effects of cortisol. Cortisol is an important stress hormone, which can have adverse health effects when levels are heightened for a prolonged time. By collecting saliva, we can look at the cortisol response on a stress task.
Participating in this study does not have prolonged negative consequences for you, as we know that the stress response on this task has a short duration.
I want to ask you whether you heard about this study before? Did you perhaps talk to persons that participated to this study?

[if yes:] What did this person tell to you?

Finally, I want to ask you not to tell anyone about the content of this study, in particular not concerning the actual study question and the presentation and arithmetic tasks. This can be very influencing to our measurements. If you know people that want to participate to this study, they are of course welcome, but tell them nothing about this study what is not standing in the advertisement or letter, as this can strongly influence the results.

22. Below, paste any other information relevant for ethical evaluation of the application e.g., advertorials, declaration of consent by external institution when participants fall under their responsibility.

Click here to enter

Do you want to earn 15 euro of 4 credits quickly?

Volunteers wanted for research to attentional processes and arithmetic skills

Are you in between 18 and 35 years of age? Then we are looking for you!

The section Health, Medical and Neuropsychology of Leiden University is looking for volunteers to participate to a study concerning attentional processes and arithmetic skills.

For this study, you will first online complete a couple of questionnaires. Afterwards, you will be invited at the Faculty of Social Sciences to complete a couple of questionnaires and to complete several tasks. Besides, we will use physiological measurement devices to measure your heart rate and skin conductance and we will ask you to provide saliva.
In total, study duration is around 2 hours.

More information? Send an e-mail to rekenonderzoek@fsw.leidenuniv.nl

supplementary information relevant for ethical review.
